# *Pseudanabaena galeata* CCNP1313—Biological Activity and Peptides Production

**DOI:** 10.3390/toxins14050330

**Published:** 2022-05-06

**Authors:** Marta Cegłowska, Karolina Szubert, Beata Grygier, Marzena Lenart, Jacek Plewka, Aleksandra Milewska, Kinga Lis, Artur Szczepański, Yuliya Chykunova, Emilia Barreto-Duran, Krzysztof Pyrć, Alicja Kosakowska, Hanna Mazur-Marzec

**Affiliations:** 1 Institute of Oceanology, Polish Academy of Sciences, Powstańców Warszawy 55, PL-81712 Sopot, Poland; akosak@iopan.gda.pl; 2 Division of Marine Biotechnology, Institute of Oceanography, University of Gdańsk, M. J. Piłsudskiego 46, PL-81378 Gdynia, Poland; karolina.szubert@phdstud.ug.edu.pl; 3 Virogenetics Laboratory of Virology, Małopolska Centre of Biotechnology, Jagiellonian University, Gronostajowa 7A, PL-30387 Cracow, Poland; beata.grygier@uj.edu.pl (B.G.); m.lenart@uj.edu.pl (M.L.); jacek.plewka@uj.edu.pl (J.P.); aleksandra.milewska@uj.edu.pl (A.M.); kinga1.lis@uj.edu.pl (K.L.); artur.szczepanski@uj.edu.pl (A.S.); y.chykunova@doctoral.uj.edu.pl (Y.C.); emilia.duran@uj.edu.pl (E.B.-D.); k.a.pyrc@uj.edu.pl (K.P.); 4 Faculty of Chemical Engineering and Technology, Cracow University of Technology, Warszawska 24, PL-31155 Cracow, Poland

**Keywords:** *Pseudanabaena galeata*, cytotoxicity, anticancer activity, antiviral activity, new peptides

## Abstract

Even cyanobacteria from ecosystems of low biodiversity, such as the Baltic Sea, can constitute a rich source of bioactive metabolites. Potent toxins, enzyme inhibitors, and anticancer and antifungal agents were detected in both bloom-forming species and less commonly occurring cyanobacteria. In previous work on the Baltic *Pseudanabaena galeata* CCNP1313, the induction of apoptosis in the breast cancer cell line MCF-7 was documented. Here, the activity of the strain was further explored using human dermal fibroblasts, African green monkey kidney, cancer cell lines (T47D, HCT-8, and A549^ACE2/TMPRSS2^) and viruses (SARS-CoV-2, HCoV-OC43, and WNV). In the tests, extracts, chromatographic fractions, and the main components of the *P. galeata* CCNP1313 fractions were used. The LC-MS/MS analyses of the tested samples led to the detection of forty-five peptides. For fourteen of the new peptides, putative structures were proposed based on MS/MS spectra. Although the complex samples (i.e., extracts and chromatographic fractions) showed potent cytotoxic and antiviral activities, the effects of the isolated compounds were minor. The study confirmed the significance of *P. galeata* CCNP1313 as a source of metabolites with potent activity. It also illustrated the difficulties in assigning the observed biological effects to specific metabolites, especially when they are produced in minute amounts.

## 1. Introduction

Cyanobacteria of the genus *Pseudanabaena* are classified to the Synechococcales order [1] and comprise almost fifty different species [2]. They live in various ecosystems, from marine and freshwater bodies to hot springs, mangrove swamps, and dry steppes [3,4,5,6,7,8,9,10]. Most of the published works have focused on the taxonomy and the presence of *Pseudanabaena* in different environments. In some studies, the effects of this cyanobacterium on other aquatic organisms have also been documented [8,11,12]. *Pseudanabaena* extracts were found to be toxic to crustaceans: *Artemia salina* [8,12], *Daphnia magna* [11], and *Ceriodaphnia dubia* [11]. They also inhibited the growth of the marine alga *Nannochloropsis* sp. LEGE Z-004 and cyanobacterium *Synechococcus nidulans* LEGE 07171 [12]. As cellular extracts were used in these studies, the active agents could not be identified. Some *Pseudanabaena* chemotypes produce known toxins, such as microcystins [13,14,15,16,17,18], anatoxin-a [18], and saxitoxin [18]; therefore, these compounds could be responsible for the observed effects. *Pseudanabaena* has also been explored as a source of metabolites with biotechnological and pharmaceutical potential [19,20,21,22]. Extracts from different strains of *Pseudanabaena* were found to be active against herpes simplex type 2 virus (HSV-2) [19], reduced oxidative stress and cellular damage in renal cells [20], and inhibited the activity of α-glucosidase [22]. Costa et al. [21] described the effects of extracts from three Portuguese *Pseudanabaena* strains on cancer cells. In another screening study, the Baltic strain, *P. galeata* CCNP1313, was cytotoxic to the breast (MCF-7) and cervical (HeLa) cancer cell lines but did not affect human dermal fibroblasts (HDF) [23].

Here, the biological activity of the Baltic *P. galeata* CCNP1313 was further explored. For this purpose, cell extracts, chromatographic fractions, and the main components of the fractions were analyzed. Cytotoxicity of the samples was tested using several cell lines, including HDF, human breast adenocarcinoma (T47D), African green monkey kidney (Vero E6), human large intestine adenocarcinoma (HCT-8), and human lung epithelial cell lines overexpressing ACE2 and TMPRSS2 (A549^ACE2/TMPRSS2^). In antiviral assays, severe acute respiratory syndrome coronavirus 2 (SARS-CoV-2), human coronavirus OC43 (HCoV-OC43), and West Nile virus (WNV) were used. The main components of the tested samples were characterized by liquid chromatography–tandem mass spectrometry (LC-MS/MS).

## 2. Results

### 2.1. Flash Chromatography and Peptide Separation

*P. galeata* CCNP1313 extract was first separated into thirty-one fractions using flash chromatography (Table S1). The fractions are marked as Fx.y, where x stands for MeOH (methanol) concentration and y is the number of the subsequent fractions eluted at x% MeOH. Additionally, four isolated peptides and four samples containing a mixture of 2–3 peptides were obtained (Table 1).

### 2.2. Bioactivity Screening

**Figure 1:**
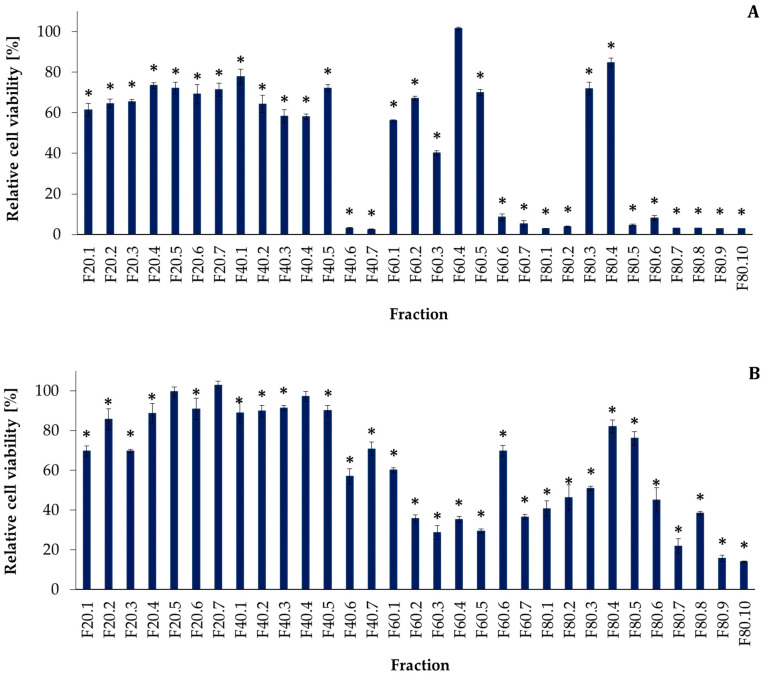
Effects of the *Pseudanabaena galeata* CCNP1313 flash chromatography fractions (200 µg mL^−1^) on the relative viability of (**A**) human breast adenocarcinoma cells (T47D) and (**B**) human dermal fibroblasts (HDF). The statistical analyses were performed with the Student *t*-test. The significance is marked with * for *p* < 0.05. The fractions are marked as Fx.y, where x stands for MeOH concentration and y is the number of the subsequent fractions eluted at x% MeOH. Data are presented as mean values with standard deviation.

First, the activity of the cell extract from *P. galeata* CCNP1313 (200 µg mL^−1^) was screened using a human breast adenocarcinoma cell line T47D and human dermal fibroblast HDF. The sample reduced the relative viability of T47D cells by 82% (SD = 1), but did not affect the primary HDF (toxicity below 1%). In the second step, the thirty-one flash chromatography fractions (200 µg mL^−1^) obtained from the extract were tested against T47D cells. Twelve of the following fractions reduced the viability of T47D by more than 90%: F40.6–F40.7, F60.6–80.2, and F80.5–80.10 (Figure 1A). The same thirty-one fractions were tested against HDF. Eleven of the fractions reduced the cell viability by more than 60%; these were fractions F60.2–F60.5, F60.7–F80.1, and F80.6–F80.10 (Figure 1B).

**Figure 2:**
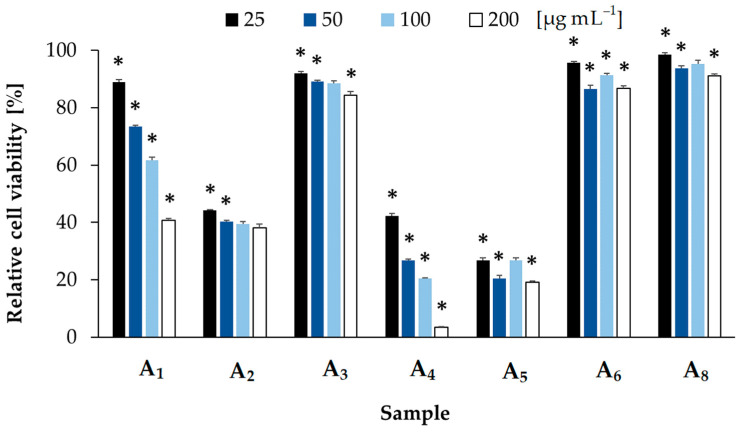
Effects of the isolated peptides (A_3_–A_5_ and A_8_) and samples containing a mixture of 2–3 peptides (A_1_, A_2_ and A_6_) from *Pseudanabaena galeata* CCNP1313 on the relative viability of the human dermal fibroblasts (HDF). The statistical analyses were performed using one-way ANOVA test. The significance is marked with * for *p* < 0.05; data are presented as mean values with standard deviation.

Then, the most active fractions were further separated and the samples with dominant components of the fractions were collected (Table 1) and tested against T47D and HDF. Neither of the samples affected the viability of T47D cells, but samples A_2_, A_4_, and A_5_ were highly cytotoxic to the HDF (Figure 2). At the lowest concentration (25 µg mL^−1^) the compounds reduced the HDF viability in a range from 56% (SD = 0; CC_50_ lower than 25 µg mL^−1^, A_2_) to 73% (SD = 1; CC_50_ lower than 25 µg mL^−1^, A_5_) (Figure 2). Sample A_1_ was less active; however, the observed effects were dose-dependent. At the highest concentration (200 µg mL^−1^), it reduced the cell viability by 60% (SD = 1). Samples A_3_, A_6_, and A_8_ did not affect the viability of HDF (toxicity below 15% at 200 µg mL^−1^) (Figure 2).

The antiviral activity of the *P. galeata* CCNP1313 cell extract was tested with the application of WNV, SARS-CoV-2, and HCoV-OC43. No cytopathic effects (CPE) were observed for HCT-8 cells infected with HCoV-OC43 and for Vero E6 cells infected with WNV. However, the extract was highly cytotoxic against Vero E6 cells infected with SARS-CoV-2. When the test was repeated with the extract roughly separated into four fractions (SPE F20–F80), the samples were not cytotoxic against Vero E6. Subsequently, reverse transcription (RT) and quantitative real-time PCR (RT-qPCR) were performed to assess the effect of the extract and SPE fractions on the viral RNA replication. The reduction in virus replication was expressed as a logarithmic reduction value (LRV) in the number of RNA copies in the infected cultures when compared to the untreated sample. The LRV for the tested viruses was equal to –1.7 (SD = 0.9) for WNV, –2.7 (SD = 0.7) for HCoV-OC43 (Figure S1), and from –0.3 (SD = 0.1) to –1.8 (SD = 0.3) for SARS-CoV-2 (Figure S2).

**Figure 3:**
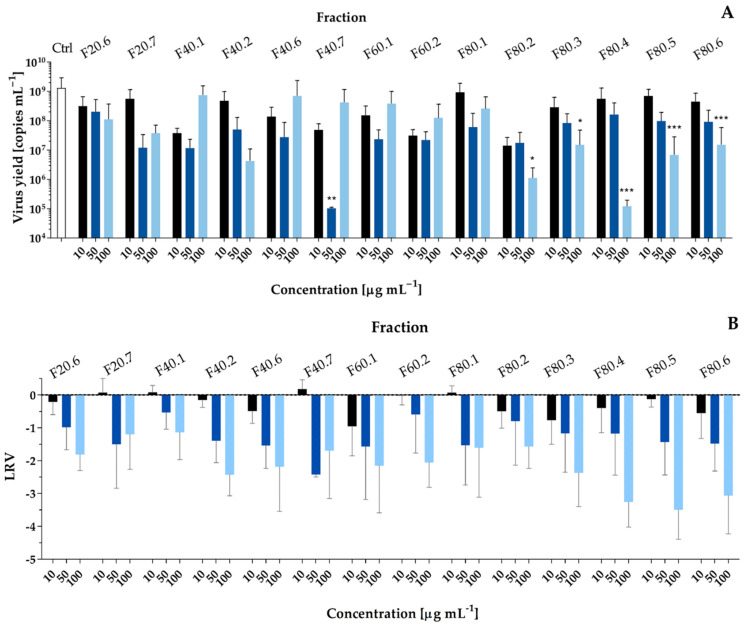
Antiviral activities of *Pseudanabaena galeata* CCNP1313 flash chromatography fractions against SARS-CoV-2 in A549^ACE2/TMPRSS2^ cells (1600 TCID50 mL^−1^; 96-h). The figure shows (**A**) the virus yield, analyzed by RT-qPCR, of the culture supernatants and (**B**) the logarithmic removal value (LRV) showing the relative decrease in the amount of the virus in the cell culture media compared to the untreated sample. Data are presented as mean values with standard deviation. The statistical analyses were performed with the Kruskal–Wallis test. The significance is marked with *** for *p* < 0.001, ** for *p* < 0.01 and * for *p* < 0.05. The fractions are marked as Fx.y, where x stands for MeOH concentration and y is the number of the subsequent fractions eluted at x% MeOH. Ctrl stands for viral control—untreated samples infected with the virus.

Next, the potential antiviral activity of the thirty-one flash chromatography fractions obtained from the *P. galeata* CCNP1313 cell extract was tested using SARS-CoV-2 and A549^ACE2/TMPRSS2^ cells. First, the CPE reduction by the fractions was assessed in a range of concentrations (1, 10, and 50 µg mL^−1^) (Table S2). Four fractions (F40.4, F40.5, F60.3, and F60.6), applied at the highest concentration, were toxic to A549^ACE2/TMPRSS2^ cells. No virus-associated CPE were observed for fractions F20.7, F80.1, and F80.3–80.5 at 50 µg mL^−1^. Additionally, fractions F20.6–F40.2, F40.6–F60.2, F80.1–F.80.3, and F80.5–F80.6 showed weak or no CPE at 10 and 50 µg mL^−1^. For these samples, the effects on SARS-CoV-2 replication were examined by RT-qPCR. Three fractions F80.4–F80.6 decreased the LRV in a dose-dependent manner, and, when applied at 100 µg mL^−1^, they reduced LRV by 3–4 LOG_10_ (Figure 3). Antiviral effects were also observed for fractions F40.2 (LRV = −2.4, SD = 0.6), F40.6 (LRV = −2.2, SD = 1.4), F40.7 (LRV = −1.7, SD = 1.5), F60.1 (LRV = −2.2, SD = 1.4), F60.2 (LRV = −2.1, SD = 0.8), F80.2 (LRV = −1.6, SD = 0.7), and F80.3 (LRV = −2.4, SD = 1.0) (Figure 3).

**Figure 4:**
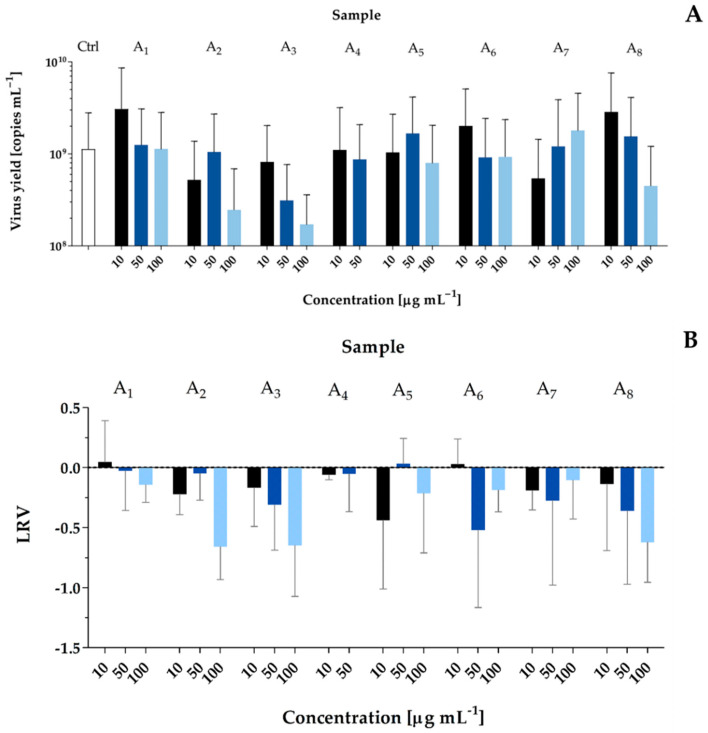
Antiviral activities of isolated peptides (A_3_–A_5_ and A_8_) and samples containing a mixture of 2–3 peptides (A_1_, A_2_ and A_6_) from *Pseudanabaena galeata* CCNP1313 against SARS-CoV-2 in A549^ACE2/TPMPRSS2^ cells (1600 TCID50 mL^−1^; 96-h). The figure shows (**A**) the virus yield, analyzed by RT-qPCR, of the cell culture supernatants and (**B**) the logarithmic removal value (LRV) showing the relative decrease in the amount of the virus in the cell culture media compared to the untreated sample. Data are presented as mean values with standard deviation. (The statistical analyses were performed with the Kruskal–Wallis test. No statistically significant differences were found). Ctrl stands for viral control—untreated samples infected with the virus.

In the case of isolated peptides (sample A_3_–A_5_ and A_8_) or samples containing a mixture of 2–3 petides (A_1_–A_2_, A_6_, and A_7_), a CPE reduction was recorded only for samples A_2_, A_3_, and A_8_ at 100 µg mL^−1^ (Table S3). These samples also had mild, but most significant effects on the SARS-CoV-2 replication (Sample A_2_ LRV = −0.7, SD = 0.3; Sample A_3_ LRV = −0.6, SD = 0.4; Sample A_8_ LRV = −0.6, SD = 0.3) (Figure 4).

Next, selected samples (at 62.5 µg mL^−1^ in the case of fractions and at 50 µg mL^−1^ in the case of isolated peptides and samples containing 2–3 peptides) were screened for potential activities against the two main proteases of SARS-CoV-2, PL^pro^ and M^pro^, which are the viral proteins often targeted by antivirals (Tables S4–S6, Figures S3–S5). Fractions eluted with 80% MeOH (F80.2–F80.6) inhibited PL^pro^ by more than 30%, with the most pronounced effects recorded for F80.5 (59%, SD = 7) (Table S4, Figure S3). The effects on M^pro^ were milder, and only for F40.7, F80.1, F80.4, and F80.5 was the inhibition slightly greater than 20% (Table S5, Figure S4). In the case of peptides or samples containing a mixture of 2–3 peptides, the strongest inhibition of PL^pro^ was observed for samples A_3_ (43%, SD = 34) and A_4_ (40%, SD = 21) (Table S6, Figure S5).

### 2.3. Structure Characterization of Compounds Present in the Active Fractions

**Figure 5:**
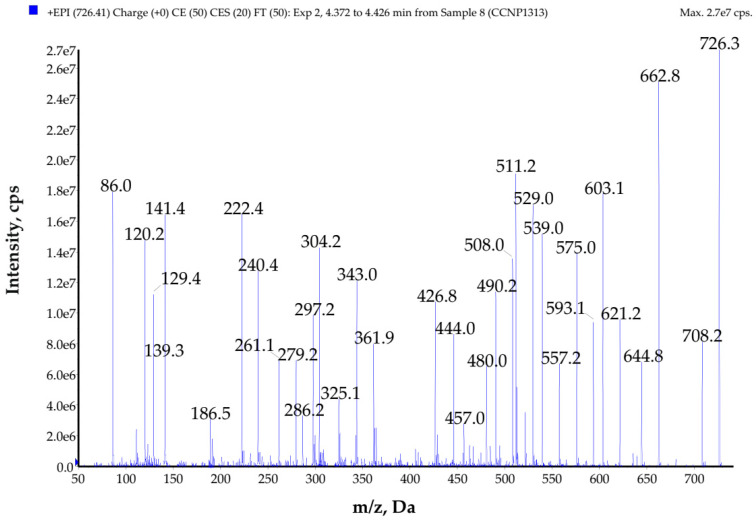
Enhanced product ion mass spectrum of the peptide named PG725 with the proposed structure Arg-MetO-Gly-Phe-Leu *-Ser, characterized based on the following ion peaks at *m*/*z* **726** [M + H]^+^, 708 [M + H − H_2_O]^+^, 662 [M + H − CH_3_SOH]^+^, 621 [M + H − Ser]^+^, 603 [M + H − Ser − H_2_O]^+^, 593 [M + H − Ser − CO]^+^, 575 [M + H − Ser − CO − H_2_O]^+^, 539 [M + H − Ser − CH_3_SOH − H_2_O]^+^, 529 [M + H − Ser − CH_3_SOH − CO]^+^, 511 [M + H − Ser − CH_3_SOH − CO − H_2_O]^+^, 508 [M + H − (Leu * + Ser)]^+^, 490 [M + H − (Leu * + Ser) − H_2_O]^+^, 480 [M + H − (Leu * + Ser) − CO]^+^, 444 [M + H − (Leu * +Ser) − CH_3_SOH]^+^, 361 [M + H − (Phe + Leu * + Ser)]^+^, 343 [M + H − (Phe + Leu * + Ser) − H_2_O]^+^, 304 [Arg + Met(O) + H]^+^, 297 [M + H − (Phe + Leu * + Ser) − CH_3_SOH]^+^, 286 [Arg + Met(O) + H − H_2_O]^+^, 279 [M + H − (Phe + Leu * + Ser) − CH_3_SOH − H_2_O]^+^, 261 [Phe + Leu * + H]^+^, 240 [Arg + Met(O) + H − CH_3_SOH]^+^, 222 [Arg + Met(O) − CH_3_SOH + H − H_2_O]^+^, 141 [Met(O) + Gly + H − CH_3_SOH]^+^, 129 Arg immonium ion, 120 Phe immonium ion, 86 Leu * immonium ion.

**Figure 6:**
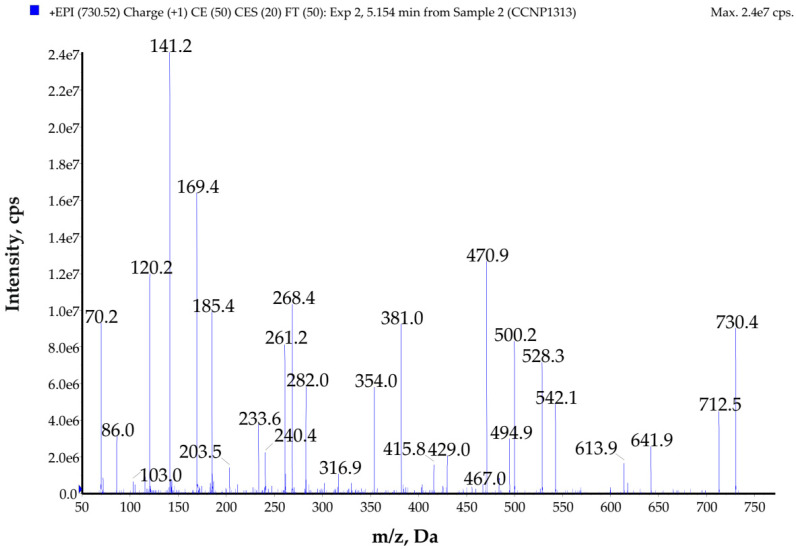
Enhanced product ion mass spectrum of galeapeptin GP729 with the proposed structure Ala-Pro-Val-Leu *-Phe-Leu *-Ala, characterized based on the following ion peaks at *m*/*z* **730** [M + H]^+^, 712 [M + H − H_2_O]^+^, 641 [M + H − Ala]^+^, 613 [M + H − Ala − CO]^+^, 542 [Pro + Val + Leu * + Phe + Leu * − CO + H]^+^, 528 [M + H − (Leu * + Ala)]^+^, 500 [M + H − (Leu * + Ala) − CO]^+^, 429 [Pro + Val + Leu * + Phe + H − CO]^+^, 381 [M + H − (Phe + Leu * + Ala)]^+^, 353 [M + H − (Phe + Leu * + Ala) − CO]^+^, 282 [Pro + Val + Leu * + H − CO]^+^, 268 [M + H − (Leu * + Phe + Leu * + Ala)]^+^, 261 [Phe + Leu * + H]^+^, 240 [M + H − (Leu * + Phe + Leu * + Ala) − CO]^+^, 233 [Phe + Leu * + H − CO]^+^, 185 [Val + Leu * + H − CO]^+^, 169 [Ala + Pro + H]^+^, 141 [Ala + Pro + H − CO]^+^, 120 Phe immonium ion, 86 Leu * immonium ion, 70 Pro immonium ion.

**Figure 7:**
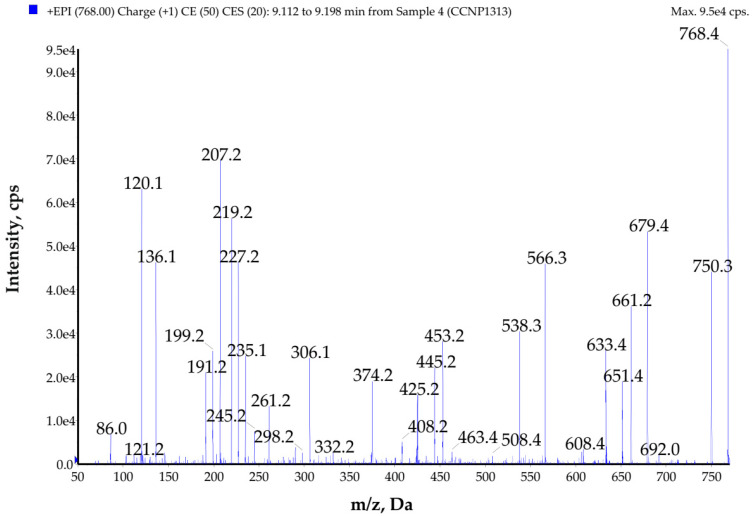
Enhanced product ion mass spectrum of galeapeptin GP767 with the proposed structure Tyr-Ala-Ala-Phe-Leu *-Leu *-Ala, characterized based on the following ion peaks at *m*/*z* **768** [M + H]^+^, 750 [M + H − H_2_O]^+^, 679 [M + H − Ala]^+^, 661 [M + H − Ala − H_2_O]^+^, 651 [M + H − Ala − CO]^+^, 633 [M + H − Ala − CO − H_2_O]^+^, 566 [M + H − (Leu * + Ala)]^+^, 538 [M + H − (Leu * + Ala) − CO]^+^, 453 [M + H − (Leu * + Leu * + Ala)]^+^, 445 [Ala + Phe + Leu * + H]^+^, 425 [M + H − (Leu * + Leu * + Ala) − CO]^+^, 374 [Phe + Leu * + Leu * + H]^+^, 306 [Tyr + Ala + Ala + H]^+^, 261 [Phe + Leu * + H]^+^, 235 [Tyr + Ala + H]^+^, 227 [Leu * + Leu * + H]^+^, 219 [Ala + Phe + H]^+^, 207 [Tyr + Ala + H − CO]^+^, 199 [Leu * + Leu * + H − CO]^+^, 191 [Ala + Phe + H − CO]^+^, 136 Tyr immonium ion, 120 Phe immonium ion, 86 Leu * immonium ion.

In order to obtain sufficient amounts of material for bioassays, only the compounds present at the highest concentrations in the active fractions were isolated. Nontargeted LC-MS/MS analyses of the fractions revealed the presence of new peptides (Table 2 and Table S1). For fourteen of the compounds, a tentative structure was suggested. De novo structure elucidation was performed manually based on collision-induced dissociation (CID) mass spectra of the precursor peptides. The spectra were reproducible and contained a high number of ion peaks. The presence of specific amino acid residues was deduced based on the immonium ion peaks and the mass differences between the subsequent high-intensity *b* ions, usually accompanied by the *a* ions. The *m*/*z* values of the pseudomolecular ions of the detected peptides ranged from 500 to 1183. The peptides contained proteinogenic residues, mainly with nonpolar side chains: Ile/Leu, Ala, Val, Phe, Pro, and Gly. The presence of Arg, Ser, Met(O) (methionine sulfoxide), Met, Thr, or Tyr was rare. In the spectra of the Met(O)-containing peptides, a characteristic loss of 64 Da units was observed (Figure 5 and Figure S7). It corresponds to methanesulfenic acid (CH_3_SOH) present in a side chain of Met(O). The structure elucidation process is demonstrated in Figure 5, Figure 6 and Figure 7, on the example of three peptides. Fragmentation spectra of the remaining peptides identified in *P. galeata* CCNP1313 are presented in the Supplementary Materials (Figures S6–S16). Because the applied method did not allow for discrimination between the isobaric ions of Leu and Ile, Leu * is used as a symbol of these residues. The structures of five of the compounds from samples A_1_, A_2_, A_5_, A_7_, and A_8_ were not elucidated. However, the presence of characteristic immonium ions and differences between ion peaks allowed us to classify them as peptides. For the dereplication of tentatively identified structures, the CyanoMetDB database [24] was searched, but no matches were found. The detected peptides with at least two Ala residues, usually at the *N*- and *C*-termini, were marked as galeapeptins (GP), while Arg and Met-containing peptides were marked as PG (*Pseudanabaena galeata* peptides). The peptides with *m*/*z* values 677, 854, 862 and 890, for which we were not able to suggest structures are marked as UP (unknown peptide).

## 3. Discussion

Among the various bioactive cyanobacterial products, compounds cytotoxic to human cancer cells were reported most frequently [25,26]. These compounds can cause inhibition of tubulin polymerization, microfilament disruption, caspase-3 activation, serine proteases inhibition or mitochondrial dysfunction, and oxidative damage [27,28]. One of the most successful cyanobacterial metabolites, dolastatin-10, was used as a lead structure to develop anticancer antibody-drug conjugates. Some of the conjugates have already been approved for cancer treatment, while others are still in clinical trials [29,30]. The antiviral activities of cyanometabolites were reported less frequently. Studies focused mainly on lectins, including cyanovirin-N (CV-N) and microvirin (MVN). These proteins bind with high affinity to oligomannose glycans of viral envelope glycoproteins gp120 [31,32]. Other antiviral agents include polysaccharides [33], peptides [34], alkaloids [35], and sulfoglycolipids [36]. Analyses of cyanobacterial metabolites performed with computational tools led to the identification of new potential antiviral agents. Deoxycylindrospermopsin showed the in silico potential to inhibit SARS-CoV-2 M^pro^ and PL^pro^ [35], while phycocyanobilin, phycoerythrobilin, phycourocilin, and folic acid from *Arthrospira* were suggested to inhibit the virus replication by interaction with the spike protein (S-protein) [37]. 

In the case of cytotoxic and antiviral activities, a significantly higher number of studies documented the effects of cyanobacterial extracts or fractionated samples than isolated metabolites with characterized structures [7,21,23,37,38]. Felczykowska et al. [23] analyzed several strains of Baltic cyanobacteria. They found that the extract from *P. galeata* CCNP1313 induced the death of the MCF-7 breast cancer cells by apoptosis, without necrotic changes or any effects on human dermal fibroblasts. Here, we showed that both the *P. galeata* CCNP1313 extract and fractions separated by the flash chromatography were active against another breast cancer cell line (T47D). As the most active fractions were separated by fractions with mild or no effects on T47D, it can be concluded that *P. galeata* CCNP1313 produces more than one metabolite with cytotoxic activity against breast cancer cells. Some of the active fractions also decreased HDF viability, but the trend in activity changes between the fractions was different to that in the case of T47D. This suggests that different agents are responsible for the activities against the two cell lines. Unfortunately, neither of the isolated peptides was found to be active in the MTT assays with T47D cells. Meanwhile, samples A_2_ (GP655, GP818, and UP853), A_4_ (PG725) and A_5_ (UP890) (Table 2) even at the lowest concentration (25 µg mL^−1^), reduced HDF viability by almost 60% (Figure 2). All isolated compounds present in the three active samples were also detected in the fractions F40.6–F60.4 (Table S1) for which a significant decrease in HDF viability was observed (Figure 1B and Figure 2). As the extract from *P. galeata* CCNP1313 was inactive against HDF, the positive results for some fractions and compounds isolated from the extract might be confusing. One of the possible reasons may be antagonistic interactions in complex samples that mask the activity of individual compounds. 

Strong effects of *P. galeata* CCNP1313 metabolites on viral replication were recorded in the case of coronaviruses HCoV-OC43 and SARS-CoV-2, and flavivirus WNV. Due to the unpredictability of their prevalence, infectious properties, and lack of effective therapeutic and preventive medicines, the threat of global infections caused by both types of viruses is constantly increasing [39,40]. Since the emergence of coronavirus disease in 2019 (COVID-19) caused by SARS-CoV-2 [41], the research on this family of pathogens has intensified. This is also the reason why, in the current work, further studies were focused on the activity of *P. galeata* CCNP1313 metabolites against SARS-CoV-2. For this purpose, the human lung epithelial cell line A549^ACE2/TMPRSS2^ overexpressing the angiotensin-converting enzyme 2 (ACE2), and the transmembrane serine proteases TMPRSS2, crucial for the SARS-CoV-2 spike protein priming was used [42,43]. A549^ACE2/TMPRSS2^ cells recapitulate the natural route of entry for the coronavirus, which enters through fusion directly on the cell surface. As a result, this makes the virus independent of the endocytic machinery and cellular cathepsins [43]. Similarly to cytotoxic effects, the antiviral activity was recorded in fractions eluted with solvent of different MeOH content. They were separated by fractions that did not induce significant effects. This result, and the fact that only some of the fractions were active in both types of assays, indicate the production of several bioactive metabolites with selective activity against specific targets. A549^ACE2/TMPRSS2^ cells infected with SARS-CoV-2 showed no or weak CPE when treated with fractions F40.2, F40.6–F60.2, and F80.3–F80.6 (Table S2). For the same fractions, the highest inhibition of viral replication was recorded. Furthermore, fractions F80.3–F80.6 showed the strongest inhibition of SARS-CoV-2 PL^pro^ (Table S4, Figure S3). This papain-like protease encoded by the viral genome is one of the proteins that are essential for viral replication [44]. The protein also dysregulates the host immune sensing [45]. Small molecules, including FDA-approved drugs, have been extensively searched for their SARS PL^pro^ inhibitory activity using high-throughput in vitro and in silico methods [44,46,47]. Although none of them has been approved yet, several PL^pro^ inhibitors are currently in clinical trials, e.g., SPI-1005 (ClinicalTrials.gov Identifier: NCT04484025) and isotretinoin (ClinicalTrials.gov Identifier: NCT04361422).

Recognition of the biological activities of fractions containing a complex mixture of metabolites is just the first step, which should lead to the identification of the hits amongst *P. galeata* CCNP1313 metabolites. Therefore, in this study, the dominant components of the most active fractions were isolated and their activities were tested. The most profound CPE reduction and the concentration-dependent inhibition of viral replication were observed for sample A_2_ containing GP655, GP818, and UP853; sample A_3_ containing GP729 (136.9 µM); and sample A_8_ containing GP1166 (85.7 µM) (Figure 4, Table S3). Based on the mass fragmentation spectra, four of the compounds (GP655, GP818, GP729, and GP1166) were classified to the same group of peptides. However, we were not able to elucidate the structure of GP1166. As this GP was also present in flash chromatography fractions showing some activity in antiviral assays (i.e., in F80.1–F80.3), this peptide may potentially be responsible for the observed effects. 

Because no matches were found in the CyanoMetDB database [24] for the compounds detected in *P. galeata* CCNP1313 samples, they are considered new structures. None of them could also be classified as a new variant of the known peptides group commonly occurring in cyanobacterial taxa. So far, the only peptides identified in *Pseudanabaena* species were microcystins [13,14,15,16,17,18]. Based on the analyses of MS/MS spectra it can be concluded that peptides detected in *P. galeata* CCNP1313 are composed of proteinogenic amino acids. In peptides with *m*/*z* 639 and 726 (named here PG638 and PG725), arginine and oxidized methionine were probably present. These two peptides have a similar structure and differ only in an additional Ser residue at the *C*-terminal part of PG725. The Met(O) present in the structure of these peptides can be an artefact formed in a nonenzymatic way during sample processing. It can also be a product of post-translational modifications generated by reactive oxygen species [48,49]. Here, the former option seems more probable as low amounts of the Met-containing variant of PG725, i.e., PG709, were also present in the extract and chromatographic fractions (Table 2, Table S1). In other compounds, mainly amino acids with hydrophobic side chains, such as those in Leu, Gly, Ala, Val, and Phe, were identified based on the immonium ions and mass differences in the MS/MS spectra. 

Numerous peptides and peptide-like structures show a broad spectrum of biological activities. Generally, they are selective in their interactions with cellular targets, do not accumulate to toxic levels in organs, and can be easily synthesized or/and modified. Their therapeutic potential as anticancer peptides (ACPs) or antiviral peptides (AVPs) has been extensively explored, frequently with the application of computational screening techniques [46,50,51,52,53]. It was found that due to negatively charged components on the surface of the cancer cell, anticancer peptides should contain cationic amino acid residues (Lys or/and Arg) to facilitate the electrostatic interaction with the cell [54]. On the other hand, the hydrophobic parts of the peptides increase their membrane penetration capability. Therefore, in ACPs, Gly, Lys, and Leu were found to be the dominant components. The activity against cancer cells can also be enhanced by the presence of Pro, Phe, and Tyr [53,55]. Hydrophobic residues and their modifications belong to the main components of several known anticancer cyanobacterial peptide-like structures such as dolastatin-10 [56], apratoxins [57], cryptophycins [58], aurilides [59], and belamide A [60]. Ala, Gly, Leu *, Phe, Pro, and Val also dominate in the structure of the peptides detected in *P. galeata* CCNP1313 samples. Although the isolated peptides did not affect T47D, the results obtained for one cancer cell line cannot eliminate the compounds from the list of potential ACPs. Peptides can also target viral or host proteins. In the case of the SARS-CoV-2 spike protein, specific residues of the receptor-binding domain can interact with AVPs, mainly through hydrogen bonding and, to a lesser degree, through hydrophobic and electrostatic interactions [51]. For these interactions, the presence of Tyr, Phe, Pro, Gly, Leu, Ala, Asn, Gln, and Cys were found to be the most important [51,52]. Considering the above findings it can be speculated, that modification of *P. galeata* CCNP1313 peptide structures by the introduction of residues likely to form hydrogen bonds with the viral proteins could enhance the activity of the compounds. But even in their natural form, the isolated peptides possess some structural elements characteristic for both ACPs and AVPs, therefor the screening of their activity against a wider array of targets could shed more light on their pharmaceutical potential.

## 4. Conclusions

The studies conducted in this work and also the results previously published by Felczykowska et al. [23] proved the significance of *P. galeata* CCNP1313 as a source of potent cytotoxic and antiviral metabolites. Unfortunately, neither of the isolated peptides was found to be responsible for the cytotoxic activity against T47D. Some antiviral effects of the peptides were observed, but they were also weaker than that recorded for chromatographic fractions. It can be speculated that the bioactive metabolites produced by *P. galeata* CCNP1313 occur in minute amounts and/or were not detected with the applied LC-MS/MS method. The other option is that the observed effects resulted from interactions of several cell components present in complex samples and could not be restored in the case of single compounds.

## 5. Materials and Methods

### 5.1. Culture Conditions

*P. galeata* CCNP1313 (GenBank accession number MN273769) [7] was grown for biomass in BG11 medium, at room temperature (22 ± 1 °C), with constant illumination (20 µM photons m^−2^ s^−1^) and aeration [61]. The biomass from 5-L cultures was harvested in the exponential growth phase by centrifugation (4000× *g*; 15 min; 4 °C) and lyophilized (Martin Christ Gefriertrocknungsanlagen, Osterode am Harz, Germany).

### 5.2. Extraction and Isolation of Metabolites

The freeze-dried cyanobacterial material (2 g portions) was extracted twice with 75% methanol in MilliQ water (25 mL × 2) by vortexing (15 min) followed by bath sonication (2 min) and centrifugation (10,000× *g*; 15 min; 4 °C). The obtained supernatants were combined and diluted in MilliQ water so that the final MeOH concentration was reduced to less than 10%. The diluted extract was applied to a preconditioned 1 g Sep-Pak Vacc tC_18_ cartridge (Waters, Milford, MA, USA) and the adsorbed substances were either eluted with 20 mL of 100% MeOH (crude extract) or with 20 mL of solvents composed of 20, 40, 60, and 80% MeOH in MilliQ water (SPE fractions). The eluates were evaporated to dryness in a centrifugal vacuum concentrator (MiVac, SP Scientific, Ipswich, UK) and tested in cytotoxicity and antiviral assays as extracts or SPE fractions.

To assess the activity of chromatographically separated fractions, 20 g of freeze-dried *P. galeata* CCNP1313 biomass was extracted twice with 500 mL of 75% MeOH as described above. The diluted extract was loaded onto a preconditioned 120 g SNAP KP-C_18_-HS flash chromatography column (Biotage, Uppsala, Sweden) using a Shimadzu HPLC system (Shimadzu Corporation, Kyoto, Japan) equipped with a photodiode array detector (PDA) and a fraction collector. A step gradient of aqueous MeOH (20–80%) was used to elute the sample components. For each of the steps of the chromatographic gradient, seven (20%–60% MeOH) or ten (80% MeOH) 40-mL fractions were collected (Table S1). Absorbance was measured at 210 and 280 nm and the flow rate was 20 mL min^−1^. Portions (5-mL) of each fraction were taken and concentrated in a centrifugal vacuum concentrator for two hours and then lyophilized. 

From selected fractions collected during flash chromatography (Table 3) the dominant components were isolated by further fractionation using the same HPLC system but different columns (Table 3). The mobile phase was composed of 5% acetonitrile in MilliQ water (A) and 100% acetonitrile (B) both with 0.1% formic acid. The conditions of three preparative chromatography runs (Prep1–Prep3) are shown in Table 3. The components of the samples containing isolated peptides or a mixture of 2–3 peptides were characterized based on their mass fragmentation spectra (Table 1). Before the assays, the samples were vacuum concentrator and lyophilized.

### 5.3. LC-MS/MS Analyses

At each step of extraction and fractionation, the contents of the samples were analyzed using a hybrid triple quadrupole/linear ion trap mass spectrometer (QTRAP5500, Applied Biosystems, Sciex, Concord, ON, Canada) coupled with an Agilent 1200 (Agilent Technologies, Waldboronn, Germany) HPLC system. The analyses were performed in a positive ion electrospray scanning mode, using a Zorbax Eclipse XDB-C_18_ column (4.6 × 150 mm; 5 µm) (Agilent Technologies, Santa Clara, CA, USA) and the same mobile phase as in preparative chromatography [61]. Spectra within the range of *m*/*z* of 500–1000 Da, and with signal intensity greater than 500,000 cps, were collected at a collision energy of 50 V. The results were processed using Analyst QS (Version 1.7.1, Applied Biosystems/MDS Analytical Technologies, Concord, ON, Canada, 2019). 

### 5.4. Cytotoxicity Assays

The evaluation of the cytotoxicity of *P. galeata* CCNP1313 was carried out in three steps. In the first step, the activity of the extract (200 µg mL^−1^) against T47D and HDF was analyzed. In the second step, the activities of thirty-one flash chromatography fractions (200 µg mL^−1^), were tested against T47D and HDF (both cell lines from the European Collection of Authenticated Cell Cultures (ECACC); Merck KGaA, Darmstadt, Germany). In the final step, the activities of samples A_1_–A_6_ and A_8_ (25–200 µg mL^−1^) against both cell lines were tested. MTT assays were performed according to Felczykowska et al. [23]. In summary, T47D cells were seeded in RPMI1640 medium (Carl Roth GmbH, Karlsruhe, Germany). HDF cells were seeded in Dulbecco’s Modified Eagle Medium (DMEM) (Gibco, Thermo Fisher Scientific Inc., Waltham, MA, USA). Both media were supplemented with fetal bovine serum (10% *v*/*v*; Merck KGaA, Darmstadt, Germany) and penicillin–streptomycin solution (50 U and 50 µg mL^−1^; Merck KGaA, Darmstadt, Germany). In the tests, the cells were seeded at 1 × 10^4^ cells per well and at each step of the test incubated at 37 °C, 5% CO_2_. The cytotoxicity of fractions dissolved in 1% DMSO was measured after incubation (24 h) with the application of a microplate reader (Spectramax i3, Molecular Devices, LLC., San Jose, CA, USA). Cell viability was calculated as the ratio of the mean absorbance value for the three replicates containing the samples to the mean absorbance of the three replicates of the corresponding solvent control and expressed as a percentage with standard deviation. Data were analyzed using the Student *t*-test or one-way ANOVA test when applicable.

### 5.5. Antiviral Activity

As in the case of cytotoxicity assays, the activities of extract, flash chromatography fractions and samples A_1_–A_8_ were analyzed.

Cell Culture. Vero E6 (ATCC: CRL-1586), HCT-8 (ATCC: CCL-244), primary human dermal fibroblasts, A549 (ATCC: CCL-185; ATCC, Manassas, VA, USA) overexpressing ACE2 and TMPRSS2 (A549^ACE2/TMPRSS2^ in-house generated [43]) were maintained in DMEM medium (Gibco, Thermo Fisher Scientific Inc., Waltham, MA, USA) supplemented with 5% v/v heat-inactivated fetal bovine serum (FBS; Gibco, Thermo Fisher Scientific Inc., Waltham, MA, USA) and penicillin-streptomycin solution (100 U mL^−1^ and 100 µg mL^−1^, respectively) (PAN Biotech GmbH, Aidenbach, Bayern, Germany). Medium for A549^ACE2/TMPRSS2^ cells was additionally supplemented with blasticidin S (10 µg mL^−1^; Sigma-Aldrich, Merck, Warsaw, Poland) and puromycin (0.5 µg mL^−1^; Sigma-Aldrich, Merck, Warsaw, Poland). 

Cytotoxicity Assays. The XTT Cell Viability Assay Kit (Biological Industries, Cromwell, CT, USA) was used according to the manufacturer’s instructions. Briefly, cells were incubated with samples at different concentrations (10–250 µg mL^−1^) for 48–120 h at 37 °C, 5% CO_2_ (Table 4). After incubation, the medium was discarded and fresh medium was overlaid on the cells, along with the activated 2,3-bis-(2-methoxy-4-nitro-5-sulfophenyl)-(2H)-tetrazolium-5-carboxanilide (XTT) solution and the cells were incubated for 2 h (37 °C, 5% CO_2_). The absorbance was measured using a microplate reader (Tecan Infinite M200; Tecan Group Ltd., Männedorf, Switzerland). Only extracts exhibiting low cytotoxicity (the viability of the treated >80% compared to the untreated samples) were further analyzed.

Cytopathic Effects Assessment. The cytopathic effects were assessed with the application of a light microscope (Evos Fluorescent Microscope, Thermo Fisher Scientific Inc., Waltham, MA, USA) 96-h post-infection (p.i.).

Virus Replication Inhibition Assays. Cells were preincubated with different concentrations of the samples (2 h, 37 °C, 5% CO_2_; Table 4). The virus infection was then performed in the presence of the samples (Table 4). After 2 h, cells were rinsed twice with PBS and a fresh medium with another portion of the tested samples was added. After 48–120 h infection (Table 4) the CPE caused by the virus in the cell monolayer was evaluated, and the culture supernatants were collected for RT-qPCR analyses of viral replication.

RNA Isolation and RT-qPCR. The isolation of viral RNA was carried out using a commercially available RNA isolation kit (Viral DNA/RNA Isolation Kit; A&A Biotechnology, Gdańsk, Poland), according to the protocol provided by the manufacturer. The isolated RNA was subjected to RT-qPCR using the GoTaq^®^ Probe 1-Step RT-qPCR System Protocol kit (Promega, Madison, WI, USA) according to the manufacturer’s instructions with the use of primers and probes (Genomed, Warsaw, Poland) listed in Table 5. Appropriate standards were prepared to evaluate the number of viral RNA molecules in the samples. The reaction was carried out in a thermal cycler (CFX96 Touch Real-197 Time PCR Detection System; Bio-Rad, Hercules, CA, USA). The obtained data are presented as LRV, showing the relative decrease in the amount of virus in the cell culture media compared to the untreated sample. Data were analyzed using the nonparametric Mann–Whitney or Kruskal–Wallis tests when applicable.

Inhibition of SARS-CoV-2 proteases. In the assays, the following reagents were used: M^pro^ and PL^pro^ from in-house production [62,63], TRIS (Bioshop, Burlington, Ontario, Canada), NaCl (Bioshop), BSA (Bioshop), DTT (Bioshop), DMSO (Sigma-Aldrich, Merck, Warsaw, Poland), synthetic peptidic M^pro^ substrate Ac-VKLQ-(AMC), synthetic peptidic Pl^pro^ substrate Ac-RLRGG-(AMC) (Enzo, New York, NY, USA). The enzymatic activities of the SARS-CoV-2 proteases M^pro^ and PL^pro^ were tested for protein at the final concentration of 160 nM in 50 mM Tris pH 8, 150 mM NaCl, 0.01% BSA, 5 mM DTT buffer, and 120 nM in 50 mM Tris pH 8, 150 mM NaCl, 0.01% BSA, 10 mM DTT buffer, respectively. 

For the assay, the protein solution (10 μL) was first loaded onto a low-volume white 96-well plate and incubated for 1 h at room temperature with 0.2 μL of inhibitors solution. The experiment was carried out in duplicate. The enzymatic reaction was started by adding 10 μL of substrate equipped with 7-amino-4-methylcoumarin (AMC) fluorophore at a final concentration of 50 μM and 2 μM, respectively. Enzymatic activity was assessed determining the changes in the fluorescence, using Tecan M200 Pro (Tecan Group Ltd., Männedorf, Switzerland). The signal was measured at 360/460 nm excitation/emission wavelengths for 90 min with 60 s intervals and with 2 s orbital shaking after each measurement. Data analyses were performed using Mathematica 12 (Wolfram, Oxfordshire, UK). 

The percentage of inhibition was calculated as a fraction of the initial velocity of the M/PL^pro^ control according to the formula:

$$100\%-\frac{\mathrm{initial}\;\mathrm{velocity}\;\mathrm{with}\;\mathrm{inhibitor}}{\mathrm{initial}\;\mathrm{velocity}\;M/\mathrm{PLpro}\;\mathrm{control}}$$

The initial velocities were calculated by fitting a straight line into datapoints from 20 to 30 using a script in Mathematica12 software. Enzymatic assays were validated using well-known PL^pro^ (GRL0617 (IC_50_: 2.1 µM) [64]) and M^pro^ inhibitors (PF-07321332, commercial name—nirmatrelvir (IC_50_: 19.2 nM), ClinicalTrials.gov Identifier: NCT05263908) (Figure S17).

## Figures and Tables

**Table 1:** The list of tested samples containing either isolated peptides or a mixture of 2–3 peptides obtained by preparative chromatography (runs P1–P3, fractions F20–80).

Sample Number	*m*/*z* and Relative Amounts * (%) of the Peptides	Preparative (P) Chromatography Fractions	Weight (mg)
A_1_	639 (75%), 862 (25%)	P1 F41	1.10
A_2_	656 (25%), 819 (60%), 854 (15%)	P1 F44–45	1.16
A_3_	730 (100%)	P1 F53–54	1.30
A_4_	726 (100%)	P1 F38 and P2 F19	1.10
A_5_	891 (100%)	P1 F87–90	1.21
A_6_	599 (32%), 716 (68%)	P2 F20–23	1.34
A_7_	677 (23%), 999 (77%)	P3 F39–40	0.70
A_8_	1167 (100%)	P3 F43–45	1.70

* The content of the compounds was assessed on the basis of the chromatographic peak area.

**Table 2:** Suggested structures and activities of peptides detected in *Pseudanabaena galeata* CCNP1313 samples ((NT) indicates not tested; (–) indicates no activity; (+) indicates weak activity; (++) indicates mild activity; (+++) indicates strong activity).

*Pseudanabaena galeata* Peptides	*m*/*z*	Suggested Chemical Structure	Sample	Cytotoxic Activity		Antiviral Activity
				T47D	HDF	
GP598	599	Ala-Leu *-Val-Leu *-Leu *-Ala	A_6_	–	–	–
PG638	639	Arg-MetO-Gly-Phe-Leu *	A_1_	–	+	–
GP655	656	Ala-Leu *-Val-Leu *-Leu *-Ala-Gly	A_2_	–	++	+
UP676	677	-	A_7_	NT	NT	–
PG709	710	Arg-Met-Gly-Phe-Leu *-Ser	-	NT	NT	NT
GP715	716	Ala-Val-Val-Phe-Leu *-Pro-Ala	A_6_	–	–	–
PG725	726	Arg-MetO-Gly-Phe-Leu *-Ser	A_4_	–	+++	–
GP729	730	Ala-Pro-Val-Leu *-Phe-Leu *-Ala	A_3_	–	–	–
GP767	768	Tyr-Ala-Ala-Phe-Leu *-Leu *-Ala	-	NT	NT	NT
GP794	795	Ala-Val-Val-Leu *-Leu *-Pro-Leu *-Ala	-	NT	NT	NT
GP818	819	Ala-Thr-Leu *-Val-Phe-Val-Val-Ala	A_2_	–	++	+
GP825	826	Ala-Pro-Val-Leu *-?-Val-Leu *-Ala	-	NT	NT	NT
GP828	829	Ala-Leu *-Val-Val-Leu *-Phe-Pro-Ala	-	NT	NT	NT
UP853	854	-	A_2_	–	++	+
UP861	862	-	A_1_	–	+	–
GP879	880	Ala-Pro-Val-?-Pro-Ala-Val-Leu *-Ala	-	NT	NT	NT
UP890	891	-	A_5_	–	+++	–
GP998	999	Ala-Pro-Val-Leu *-Ala-Phe-Val-Val-Leu *-Ala	A_7_	NT	NT	NT
GP1166	1167	-	A_8_	–	–	+

* Because the applied method did not allow for discrimination between the isobaric ions of Leu and Ile, Leu * is used as a symbol of these residues.

**Table 3:** Chromatographic conditions used for the separation of the main components of the flash chromatography fractions obtained from the *Pseudanabaena galeata* CCNP1313 extract.

	Prep1	Prep2	Prep3
	Flash Chromatography Fractions		
	F40%.7–F60%.3	F40%.2 and F40%.6	F80%.1–F80%.3
Precolumn	SecurityGuard Prep Cartridge C_12_ (15 × 30 mm; 90 Å; 4 µm) (Phenomenex, Torrence, CA, USA)	ReproSil-Pur Basic C_18_ (5 × 4.6 mm; 100 Å; 5 µm) (Dr. Maisch GmbH, Ammerbuch, Germany)	
Column	Jupiter Proteo C_12_ (250 × 21.2 mm; 90 Å; 4 µm) (Phenomenex, Torrence, CA, USA) Flow rate: 15 mL min^−1^	ReproSil-Pur Basic C_18_ (250 × 4.6 mm; 100 Å; 5 µm) (Dr. Maisch GmbH, Ammerbuch, Germany) Flow rate: 1.5 mL min^−1^	
Gradient	From 10% B to 100% B in 35 min	From 20% B to 100% B in 35 min	From 15% B to 100% B in 70 min
Fraction volume	3 mL per vial	1.5 mL per vial	1.5 mL per vial

**Table 4:** Infection models and tested *Pseudanabaena galeata* CCNP1313 samples.

Virus	Cells	Infection Culture Condition	Samples
**WNV** strain Hungary 578/10 TCID_50_ = 400 mL^−1^	Vero E6	37 °C, 5% CO_2_, 48 h	Extract; 250 µg mL^−1^
**HCoV-OC43** ATCC: VR-1558 TCID_50_ = 400 mL^−1^	HCT-8	32 °C, 5% CO_2_, 96–120 h	Extract; 250 µg mL^−1^
**SARS-CoV-2** TCID_50_ = 400 mL^−1^ PL_P18 [GISAID Clade G, Pangolin lineage B.1] (accession numbers for the GISAID database: EPI_ISL_451979)	Vero E6	37 °C, 5% CO_2_, 72 h	F20, F40, F60, F80; 50 µg mL^−1^
**SARS-CoV-2** TCID_50_ = 1600 mL^−1^ PL_P18 [GISAID Clade G, Pangolin lineage B.1] (accession numbers for the GISAID database: EPI_ISL_451979)	A549^ACE2/TMPRSS2^	37 °C, 5% CO_2_, 96 h	Flash chromatography fractions; samples A_1_–A_8_; 10, 50, 100 µg mL^−1^

**Table 5:** Oligonucleotides used in RT-qPCR.

Virus	Oligonucleotides	Sequence (5′ -> 3′)
**ZIKV**	Primer 5′ (Forward)	TTGGTCATGATACTGCTGATTGC
	Primer 3′ (Reverse)	CCTTCCACAAAGTCCCTATTGC
	Probe	6-FAM-CGGCATACAGCATCAGGTGCATAGGAG-TAMRA
**WNV**	Primer 5′ (Forward)	CGGAAGTyGrGTAkACGGTGCTG
	Primer 3′ (Reverse)	CGGTwyTGAGGGCTTACrTGG
	Probe	6-FAM-wCCCCAGGwGGACTG-BHQ1
**HCoV-OC43**	Primer 5′ (Forward)	AGCAACCAGGCTGATGTCAATACC
	Primer 3′ (Reverse)	AGCAGACCTTCCTGAGCCTTCAAT
	Probe	6-FAM-TGACATTGTCGATCGGGACCCAAGTA-TAMRA
**SARS-CoV-2**	Primer 5′ (Forward)	CACATTGGCACCCGCAATC
	Primer 3′ (Reverse)	GAGGAACGAGAAGAGGCTTG
	Probe	6-ACTTCCTCAAGGAACAACATTGCCA-BHQ-1

## Data Availability

Not applicable.

## Supplementary Materials

The following supporting information can be downloaded at: https://www.mdpi.com/article/10.3390/toxins14050330/s1. Table S1: The content of the *Pseudanabaena galeata* CCNP1313 flash chromatography fractions. Figure S1: Antiviral activities of the *Pseudanabaena galeata* CCNP1313 crude extract against human coronavirus OC43 (HCoV-OC43) in HCT-8 cells and West Nile Virus (WNV) in Vero E6 cells. The figure shows (A) virus yield, analyzed by RT-qPCR, of cell culture supernatants and (B) logarithmic removal value (LRV) showing the relative decrease in the amount of the virus in the cell culture media compared to the untreated samples. Ctrl stands for viral control—untreated samples infected with the virus. Data are presented as mean values with standard deviation; the statistical analyses were performed with the Mann—Whitney test. The significance is marked with * for *p* < 0.05. Figure S2: Antiviral activities of the *Pseudanabaena galeata* CCNP1313 solid-phase extraction (SPE) fractions against severe acute respiratory syndrome coronavirus 2 (SARS-CoV-2) in Vero E6 cells. The figure shows (A) virus yield, analyzed by RT-qPCR, of cell culture supernatants and (B) the logarithmic removal value (LRV) showing the relative decrease in the amount of the virus in the cell culture media compared to the untreated sample. Ctrl stands for viral control—untreated samples infected with the virus. Data are presented as mean values with standard deviation; the statistical analyses were performed with the Kruskal–Wallis test. The significance is marked with * for *p* < 0.05. Table S2: The cytopathic effects (CPE) of the *Pseudanabaena galeata* CCNP1313 flash chromatography fractions after SARS-CoV-2 infection of the A549^ACE2/TMPRSS2^ cell line. The CPE was assessed microscopically after 96-h from the infection (TCID_50_ = 1600 mL^−1^ based on the following scale: (+++) indicates no reduction of infection-induced CPE, comparable to the positive control; (++) indicates 20–40% reduction of infection-induced CPE; (+) indicates 50–70% reduction of infection-induced CPE; (−) indicates maximal infection reduction, comparable to the mock-infected samples (+/−) indicates an uncertain CPE; (tox) indicates toxicity). Table S3: The cytopathic effects (CPE) of isolated peptides (A_3_–A_5_ and A_8_) and samples containing a mixture of 2–3 peptides (A_1_, A_2_ and A_6_) from *Pseudanabaena galeata* CCNP1313 after SARS-CoV-2 infection of the A549^ACE2/TMPRSS2^ cell line. The CPE was assessed microscopically after 96-h from the infection (TCID_50_ = 1600 mL^−1^ based on the following scale: (+++) indicates no reduction of infection-induced CPE, comparable to the positive control; (++) indicates 20–40% reduction of infection-induced CPE; (+) indicates 50–70% reduction of infection-induced CPE; (−) indicates maximal infection reduction, comparable to the mock-infected samples. (NT) indicates not tested). Table S4: The effects of selected *Pseudanabaena galeata* CCNP1313 flash chromatography fractions on the activity of SARS-CoV-2 PL^Pro^ protease. Data are presented as mean values with standard deviation. Figure S3: The effects of selected *Pseudanabaena galeata* CCNP1313 flash chromatography fractions on the activity of SARS-CoV-2 PL^Pro^ protease. The fractions are marked as Fx.y, where x stands for MeOH concentration and y is the number of the subsequent fractions eluted at x% MeOH. Data are presented as mean values. Table S5: The effects of selected *Pseudanabaena galeata* CCNP1313 flash chromatography fractions on the activity of SARS-CoV-2 M^Pro^ protease. inhibition fractions. Data are presented as mean values with standard deviation. Figure S4: The effects of selected *Pseudanabaena galeata* CCNP1313 flash chromatography fractions on the activity of SARS-CoV-2 M^Pro^ protease. The fractions are marked as Fx.y, where x stands for MeOH concentration and y is the number of the subsequent fractions eluted at x% MeOH. Data are presented as mean values. Table S6: The effects of isolated peptides and samples containing a mixture of 2–3 peptides from *Pseudanabaena galeata* CCNP1313 on the activity of SARS-CoV-2 PL^Pro^ protease. Data are presented as mean values with standard deviation. Figure S5: The effects of isolated peptides and samples containing a mixture of 2–3 peptides from *Pseudanabaena galeata* CCNP1313 on the activity of SARS-CoV-2 PL^Pro^ protease. Figure S6: Enhanced product ion mass spectrum of galeapeptin GP598 with the proposed structure Ala-Leu *-Val-Leu *-Leu *-Ala, characterized based on the following ion peaks at *m*/*z* 599 [M + H]^+^, 581 [M + H − H_2_O]^+^, 510 [M + H − Ala *]^+^, 483 [M + H − Ala − CO]^+^, 397 [M + H − (Leu * + Ala]^+^/[Ala + Leu * + Val + Leu * + H]^+^, 369 [Ala + Leu * + Val + Leu * + H − CO]^+^, 284 [Ala + Leu * + Val + H]^+^, 266 [Ala + Leu * + Val − H_2_O + H]^+^, 256 [Ala + Leu * + Val − CO + H]^+^, 213 [Leu * + Val + H]^+^, 185 [Ala + Leu * + H]^+^/[Leu * + Val − CO + H]^+^, 157 [Leu * + Ala − CO + H]^+^, 86 Leu * immonium ion, 72 Val immonium ion. Figure S7: Enhanced product ion mass spectrum of a peptide named PG638 with the proposed structure Arg-MetO-Gly-Phe-Leu *, characterized based on the following ion peaks at *m*/*z* 639 [M + H]^+^, 621 [M + H − H_2_O]^+^, 575 [M + H − CH_3_SOH]^+^, 508 [M + H − Leu *]^+^, 490 [M + H − Leu * − H_2_O]^+^, 480 [M + H − Leu * − H_2_O]^+^, 444 [M + H − Leu * − CH_3_SOH]^+^, 426 [M + H − Leu * − CH_3_SOH − H_2_O]^+^, 416 [M + H − Leu * − CH_3_SOH − CO]^+^, 398 [M + H − Leu * − CH_3_SOH − H_2_O − CO]^+^, 361 [M + H − (Phe + Leu *)]^+^, 343 [M + H − (Phe + Leu *) − H_2_O]^+^, 304 [Arg + Met(O) + H]^+^, 297 [M + H − CH_3_SOH − (Phe + Leu *)]^+^, 286 [Arg + Met(O) + H − H_2_O]^+^, 279 [M + H − CH_3_SOH − (Phe + Leu *) − H_2_O]^+^, 240 [Arg + Met(O) + H − CH_3_SOH]^+^, 222 [Arg + Met(O) + H − CH_3_SOH − H_2_O]^+^, 141 [Met(O) + Gly + H − CH_3_SOH]^+^, 129 Arg immonium ion, 120 Phe immonium ion, 86 Leu * immonium ion. Figure S8: Enhanced product ion mass spectrum of the galeapeptin GP655 with the proposed structure Ala- Leu *-Val-Leu *-Leu *-Ala-Gly, characterized based on the following ion peaks at *m*/*z* 656 [M + H]^+^, 638 [M + H − H_2_O]^+^, 581 [M + H − Gly]^+^, 563 [M + H − Gly − H_2_O]^+^, 553 [M + H − Gly − CO]^+^, 510 [Ala + Leu * + Val + Leu * + Leu * + H]^+^, 492 [Ala + Leu * + Val + Leu * + Leu * + H − H_2_O]^+^, 482 [Ala + Leu * + Val + Leu * + Leu * + H − CO]^+^, 397 [M + H − (Leu * + Ala + Gly)]^+^/[Ala + Leu * + Val + Leu * + H]^+^, 369 [Ala + Leu * + Val + Leu * + H − CO]^+^, 284 [Ala + Leu * + Val + H]^+^, 266 [Ala + Leu * + Val + H − H_2_O]^+^, 256 [Ala + Leu * + Val + H − CO]^+^, 213 [Leu * + Val + H]^+^, 195 [Leu * + Val + H − H_2_O]^+^, 185 [Ala + Leu * + H]^+^/[Leu * + Val + H − CO]^+^, 167 [Ala + Leu * + H − H_2_O]^+^, 157 [Leu * + Ala + H − CO]^+^, 86 Leu * immonium ion, 72 Val immonium ion. Figure S9: Enhanced product ion mass spectrum of peptide a peptide named PG709 with the proposed structure Arg-Met-Gly-Phe-Leu *-Ser, characterized based on the following ion peaks at *m*/*z* 710 [M + H]^+^, 692 [M + H − H_2_O]^+^, 682 [M + H − CO]^+^, 605 [M + H − Ser]^+^, 587 [M + H − Ser − H_2_O]^+^, 577 [M + H − Ser − CO]^+^, 559 [M + H − Ser − CO − H_2_O]^+^, 492 [M + H − (Leu * + Ser)]^+^, 474 [M + H − (Leu * + Ser) − H_2_O]^+^, 464 [M + H − (Leu * + Ser) − CO]^+^, 446 [M + H − (Leu * + Ser) − H_2_O − CO]^+^, 345 [Arg + Met + Gly + H]^+^, 327 [Arg + Met + Gly + H − H_2_O]^+^, 270 [Arg + Met + H − H_2_O]^+^, 129 Arg immonium ion, 120 Phe immonium ion, 86 Leu * immonium ion. Figure S10: Enhanced product ion mass spectrum of galeapeptin GP715 with the proposed structure Ala-Val-Val-Phe-Leu *-Pro-Ala, characterized based on the following ion peaks at *m*/*z* 716 [M + H]^+^, 627 [M + H − Ala]^+^, 599 [M + H − Ala − CO]^+^, 530 [M + H − (Pro + Ala)]^+^, 502 [M + H − (Pro + Ala) − CO]^+^, 460 [Val + Val + Phe + Leu * + H]^+^, 432 [Val + Val + Phe + Leu * + H − CO]^+^, 417 [M + H − (Leu * + Pro + Ala)]^+^, 389 [M + H − (Leu * + Pro + Ala) − CO]^+^, 346 [Val + Val + Phe + H]^+^, 318 [Val + Val + Phe + H − CO]^+^, 270 [Ala + Val + Val + H]^+^, 261 [Phe + Leu * + H]^+^, 247 [Val + Phe + H]^+^, 233 [Phe + Leu * + H − CO]^+^, 219 [Val + Phe + H − CO]^+^, 211 [Leu * + Pro + H]^+^, 199 [Val + Val + H]^+^, 171 [Ala + Val + H]^+^, 143 [Ala + Val + H − CO]^+^, 120 Phe immonium ion, 86 Leu * immonium ion, 72 Val immonium ion, 70 Pro immonium ion. Figure S11: Enhanced product ion mass spectrum of galeapeptin G794 with the proposed structure Ala-Val-Val- Leu *-Leu *-Pro-Leu *-Ala, characterized based on the following ion peaks at *m*/*z* 795 [M + H]^+^, 706 [M + H − Ala]^+^, 678 [M + H − Ala − CO]^+^, 593 [M + H − (Leu * + Ala)]^+^, 565 [M + H − (Leu * + Ala) − CO]^+^, 496 [M + H − (Pro + Leu * + Ala)]^+^, 468 [M + H − (Pro + Leu * + Ala) − CO]^+^, 383 [Ala + Val + Val + Leu * + H]^+^, 355 [Ala + Val + Val + Leu * + H − CO]^+^, 324 [Leu * + Leu * + Pro + H]^+^, 270 [Ala + Val + Val + H]^+^, 211 [Leu * + Pro + H]^+^, 199 [Leu * + Leu * + H − CO]^+^, 183 [Leu * + Pro + H − CO]^+^, 171 [Ala + Val + H]^+^, 143 [Ala + Val + H − CO]^+^, 86 Leu * immonium ion, 72 Val immonium ion, 70 Pro immonium ion. Figure S12: Enhanced product ion mass spectrum of galeapeptin GP818 with the proposed structure Ala-Thr-Leu *-Val-Phe-Val-Val-Ala, characterized based on the following ion peaks at *m*/*z* 819 [M + H]^+^, 801 [M + H − H_2_O]^+^, 730 [M + H − Ala]^+^, 712 [M + H − Ala − H_2_O]^+^, 702 [M + H − Ala − CO]^+^, 684 [M + H − Ala − H_2_O − CO]^+^, 631 [M + H − (Val + Ala)]^+^, 613 [M + H − (Val + Ala) − H_2_O]^+^, 603 [M + H − (Val + Ala) − CO]^+^, 532 [M + H − (Val + Val + Ala)]^+^, 514 [M + H − (Val + Val + Ala) − H_2_O]^+^, 504 [M + H − (Val + Ala) − CO]^+^, 367 [M + H − (Phe − Val + Val + Ala) − H_2_O]^+^, 213 [Leu * + Val + H]^+^, 185 [Ala + Leu * + H]^+^, 157 [Ala + Leu * + H − CO]^+^, 171 [Val + Val + H − CO]^+^, 120 Phe immonium ion, 86 Leu * immonium ion, 74 Thr immonium ion, 72 Val immonium ion. Figure S13: Enhanced product ion mass spectrum of galeapeptin GP825 with the proposed partially elucidated structure Ala-Pro-Val-Leu *-?-Val-Leu *-Ala, characterized based on the following ion peaks at *m*/*z* 826 [M + H]^+^, 808 [M + H − H_2_O]^+^, 737 [M + H − Ala]^+^, 624 [M + H − (Leu * + Ala)]^+^, 596 [M + H − (Leu * + Ala) − CO]^+^, 525 [M + H − (Val + Leu * + Ala)]^+^, 497 [M + H − (Val + Leu * + Ala) − CO]^+^, 381 [Ala + Pro + Val + Leu * + H]^+^, 353 [Ala + Pro + Val + Leu * + H − CO]^+^, 268 [Ala + Pro + Val + H]^+^, 169 [Ala + Pro + H]^+^, 141 [Ala + Pro + H − CO]^+^, 120 Phe immonium ion, 86 Leu * immonium ion. Figure S14: Enhanced product ion mass spectrum of galeapeptin GP828 with the proposed structure Ala-Leu *-Val-Val-Leu *-Phe-Pro-Ala, characterized based on the following ion peaks at *m*/*z* 829 [M + H]^+^, 740 [M + H − Ala]^+^, 712 [M + H − Ala − CO]^+^, 643 [M + H − (Pro + Ala)]^+^, 625 [M + H − (Pro + Ala) − H_2_O]^+^, 615 [M + H − (Pro + Ala) − CO]^+^, 572 [M + H − Ala − (Pro + Ala)]^+^, 544 [M + H − Ala − (Pro + Ala) − CO]^+^, 496 [M + H − (Pro + Ala)]^+^, 468 [M + H − (Pro + Ala) − CO]^+^, 383 [Ala + Leu * + Val + Val + H]^+^, 355 [Ala + Leu * + Val + Val + H − CO]^+^, 312 [Leu * + Val + Val + H]^+^, 284 [Ala + Leu * + Val + H]^+^, 213 [Leu * + Val + H]^+^, 185 [Leu * + Val + H − CO]^+^/ [Ala + Leu * + H]^+^, 199 [Val + Val + H]^+^, 157 [Ala + Leu * + H − CO]^+^, 171 [Val + Val + H − CO]^+^, 120 Phe immonium ion, 86 Leu * immonium ion, 72 Val immonium ion, 70 Pro immonium ion. Figure S15: Enhanced product ion mass spectrum of galeapeptin GP879 with the proposed partially elucidated structure Ala-Pro-Val-?-Pro-Ala-Val-Leu *-Ala, characterized based on the following ion peaks at *m*/*z* 880 [M + H]^+^, 862 [M + H − H_2_O]^+^, 791 [M + H − Ala]^+^, 773 [M + H − Ala − H_2_O]^+^, 678 [M + H − (Leu * + Ala)]^+^, 660 [M + H − (Leu * + Ala) − H_2_O]^+^, 650 [M + H − (Leu * + Ala) − CO]^+^, 632 [M + H − (Leu * + Ala) − H_2_O − CO]^+^, 579 [M + H − (Val + Leu * + Ala)]^+^, 561 [M + H − (Val + Leu * + Ala) − H_2_O]^+^, 551[M + H − (Val + Leu * + Ala) − H_2_O − CO]^+^, 508 [M + H − (Ala + Val + Leu * + Ala)]^+^, 411 [M + H − (Pro + Ala + Val + Leu * + Ala)]^+^, 393 [M + H − (Pro + Ala + Val + Leu * + Ala) − H_2_O]^+^, 383 [M + H − (Pro + Ala + Val + Leu * + Ala) − CO]^+^, 381 [Ala + Pro + Val + Leu * + H]^+^, 365 [M + H − (Pro + Ala + Val + Leu * + Ala) − H_2_O − CO]^+^, 353 [Ala + Pro + Val + Leu * + H − CO]^+^, 268 [Ala + Pro + Val + H]^+^, 169 [Ala + Pro + H]^+^, 141 [Ala + Pro + H − CO]^+^, 120 Phe immonium ion, 86 Leu * immonium ion. Figure S16: Enhanced product ion mass spectrum of galeapeptin GP998 with the proposed structure Ala-Pro-Val-Leu *-Ala-Phe-Val-Val-Leu *-Ala, characterized based on the following ion peaks at *m*/*z* 999 [M + H]^+^, 981 [M + H − H_2_O]^+^, 910 [M + H − Ala *]^+^, 882 [M + H − Ala − CO]^+^, 797 [M + H − (Leu * + Ala)]^+^, 769 [M + H − (Leu * + Ala) − CO]^+^, 698 [M + H − (Val + Leu * + Ala)]^+^, 599 [M + H − (Val + Val + Leu * + Ala)]^+^, 571 [M + H − (Val + Val + Leu * + Ala) − CO]^+^, 530 [Val + Leu * + Ala + Phe + Val + H]^+^, 452 [Ala + Pro + Val + Leu * + Ala + H]^+^, 431 [Val + Leu * + Ala + Phe + H]^+^, 417 [Ala + Phe + Val + Val +H]^+^, 403 [Val + Leu * + Ala + Phe + H − CO]^+^, 381 [Ala + Pro + Val + Leu * + H]^+^, 353 [Ala + Pro + Val + Leu * + H - CO]^+^, 332 [Leu * + Ala + Phe + H]^+^, 268 [Ala + Pro + Val + H]^+^, 219 [Ala + Phe + H]^+^, 213 [Val + Leu * + H]^+^, 199 [Val + Val + H]^+^, 185 [Val + Leu * − CO + H]^+^, 171 [Val + Val + H − CO]^+^, 169 [Ala + Pro + H]^+^, 141 [Ala + Pro + H − CO]^+^, 120 Phe immonium ion, 86 Leu * immonium ion, 72 Val immonium ion. Figure S17: Positive controls and IC_50_ values determination for enzymatic assays (A) M^pro^ (IC_50_ for PF-07321332 a well-known M^pro^ inhibitor, commercial name nirmatrelvir with reported literature IC_50_ value of 19.2 nM) and (B) PL^pro^ (IC_50_ determination for GRL-0617, a well-known PL^pro^ inhibitor with reported literature IC_50_ value of 2.1 µM).
