# Supplementary material for: Pseudanabaena galeata CCNP1313—Biological Activity and Peptides Production
Source: Toxins (Basel). 2022 May 6;14(5):330. doi: 10.3390/toxins14050330 (PMC9146944; doi:10.3390/toxins14050330)
Supplement: Supplementary file 1 [file toxins-14-00330-s001.zip › toxins-1711445-SM.pdf]

toxins

MDPI

Article

Pseudanabaena galeata CCNP1313 biological activity and peptides production

Marta Cegłowska, Karolina Szubert, Beata Grygier, Marzena Lenart, Jacek Plewka, Aleksandra Milewska, Kinga Lis, Artur Szczepański, Yuliya Chykunova, Emilia Barreto-Duran, Krzysztof Pyrć, Alicja Kosakowska and Hanna Mazur-Marzec

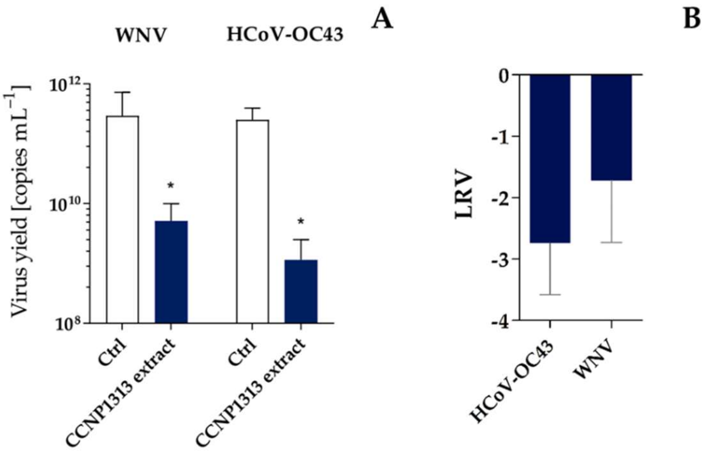

Figure S1. Antiviral activities of the *Pseudanabaena galeata* CCNP1313 crude extract against human coronavirus OC43 (HCoV-OC43) in HCT-8 cells and West Nile Virus (WNV) in Vero E6 cells. The figure shows (**A**) virus yield, analysed by RT-qPCR, of cell culture supernatants and (**B**) logarithmic removal value (LRV) showing the relative decrease in the amount of the virus in the cell culture media compared to the untreated samples. Ctrl stands for viral control—untreated samples infected with the virus. Data are presented as mean values with standard deviation; the statistical analyses were performed with the Mann–Whitney test. The significance is marked with * for $p < 0.05$.

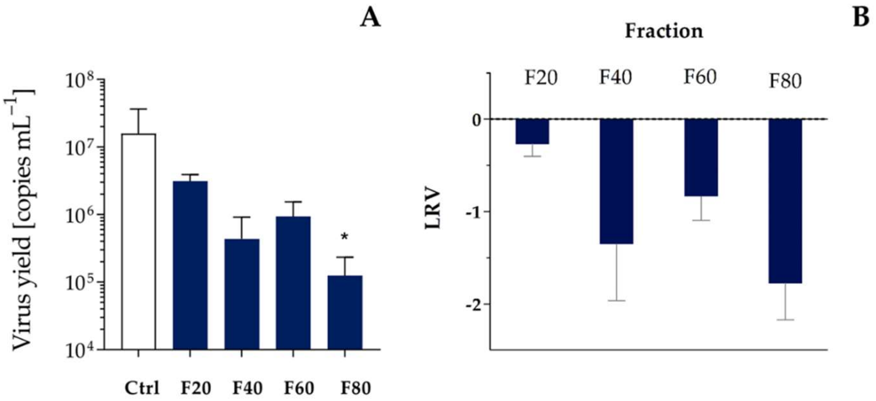

Figure S2. Antiviral activities of the *Pseudanabaena galeata* CCNP1313 solid-phase extraction (SPE) fractions against severe acute respiratory syndrome coronavirus 2 (SARS-CoV-2) in Vero E6 cells. The figure shows (**A**) virus yield, analysed by RT-qPCR, of cell culture supernatants and (**B**) the logarithmic removal value (LRV) showing the relative decrease in the amount of the virus in the cell culture media compared to the untreated sample. Ctrl stands for viral control—untreated samples infected with the virus. Data are presented as mean values with standard deviation; the statistical analyses were performed with the Kruskal–Wallis test. The significance is marked with * for $p < 0.05$.

Table S2. The cytopathic effects (CPE) of the *Pseudanabaena galeata* CCNP1313 flash chromatography fractions after SARS-CoV-2 infection of the A549$^{ACE2/TMPRSS2}$ cell line. The CPE was assessed microscopically after 96-h from the infection ($TCID_{50}$ = 1600 mL^{-1} based on the following scale: (+++) indicates no reduction of infection-induced CPE, comparable to the positive control; (++)indicates 20-40% reduction of infection-induced CPE; (+) indicates 50–70% reduction of infection-induced CPE; (–) indicates maximal infection reduction, comparable to the mock-infected samples (+/–) indicates an uncertain CPE; (tox) indicates toxicity).

**Flash chromatography fraction** *	**Extract concentration**					
	1 μg mL^{-1}		10 μg mL^{-1}		50 μg mL^{-1}	
	**Repetition**					
	I	II	I	II	I	II
F20.1	+++	+++	++	++	+	+
F20.2	+	+++	++	++	+	++
F20.3	+++	+++	++	++	+++	++
F20.4	+++	+++	++	++	+	-
F20.5	+++	+++	+	+	+	+
F20.6	+++	+++	+	+	-	+/-
F20.7	+++	++	-	+	-	-
F40.1	++	+++	+	+	+	+
F40.2	+	++	+	+	-	+/-
F40.3	+++	+++	++	+	+/-	+
F40.4	+++	++	+	+	tox	tox
F40.5	+++	++	+	+	+/-	tox
F40.6	+	++	+	-	+/-	-
F40.7	+	++	+	+	+/-	+/-
F60.1	+	+	-	-	+/-	-
F60.2	+++	++	+	+/-	-	+/-
F60.3	++	++	+	+	+ (tox)	++ (tox)
F60.4	++	+	+/-	+	+	++
F60.5	++	++	+	++	+	+
F60.6	++	++	+	+	tox	++ (tox)
F60.7	++	++	+++	+	+	++
F80.1	++	++	+	+	-	-
F80.2	++	++	+	+	+	+/-
F80.3	+	++	+	+	-	-
F80.4	+	++	++	++	-	-
F80.5	+	++	+	+	-	-
F80.6	++	++	+	+	+/-	+
F80.7	++	++	++	++	+	+
F80.8	+++	+++	++	+	+	+/-
F80.9	++	++	+	+	+/-	+
F80.10	++	++	+	++	++	-

* The fractions are marked as Fx.y, where x stands for MeOH concentration and y is the number of the subsequent fractions eluted at x% MeOH.

Table S3. The cytopathic effects (CPE) of isolated peptides (A_3–A_5 and A_8) and samples containing a mixture of 2–3 peptides (A_1, A_2 and A_6) from *Pseudanabaena galeata* CCNP1313 after SARS-CoV-2 infection of the $A549^{ACE2/TMPRSS2}$ cell line. The CPE was assessed microscopically after 96-h from the infection ($TCID_{50}$ = 1600 mL^{-1} based on the following scale: (+++) indicates no reduction of infection-induced CPE, comparable to the positive control; (++) indicates 20-40% reduction of infection-induced CPE; (+) indicates 50–70% reduction of infection-induced CPE; (–) indicates maximal infection reduction, comparable to the mock-infected samples. (NT) indicates not tested).

Sample	Sample concentration					
	10 µg mL^{-1}		50 µg mL^{-1}		100 µg mL^{-1}	
	Repetition					
	I	II	I	II	I	II
A_1	+++	+++	++	++	+++	+
A_2	+++	+++	++	++	+	+
A_3	++	+++	+	++	-	+
A_4	+++	+++	++	++	**NT**	**NT**
A_5	+++	+++	++	+++	+	++
A_6	+++	+++	+++	+++	++	+++
A_7	+++	+++	+++	+++	+++	+++
A_8	+++	+++	++	+++	+	+

Table S4. The effects of selected *Pseudanabaena galeata* CCNP1313 flash chromatography fractions on the activity of SARS-CoV-2 PL^{Pro} protease. Data are presented as mean values with standard deviation.

Fraction*	Initial velocity (mAU min^{-1})	SD	Inhibition (%)
PL^{Pro}	615	1	0
F20.6	55	5	10
F20.7	512	28	17
F40.2	531	20	14
F40.6	423	38	31
F60.2	491	16	20
F80.2	413	40	33
F80.3	33	36	46
F80.4	316	19	49
F80.5	251	7	59
F80.6	278	10	55

* The fractions are marked as Fx.y, where x stands for MeOH concentration and y is the number of the subsequent fractions eluted at x% MeOH.

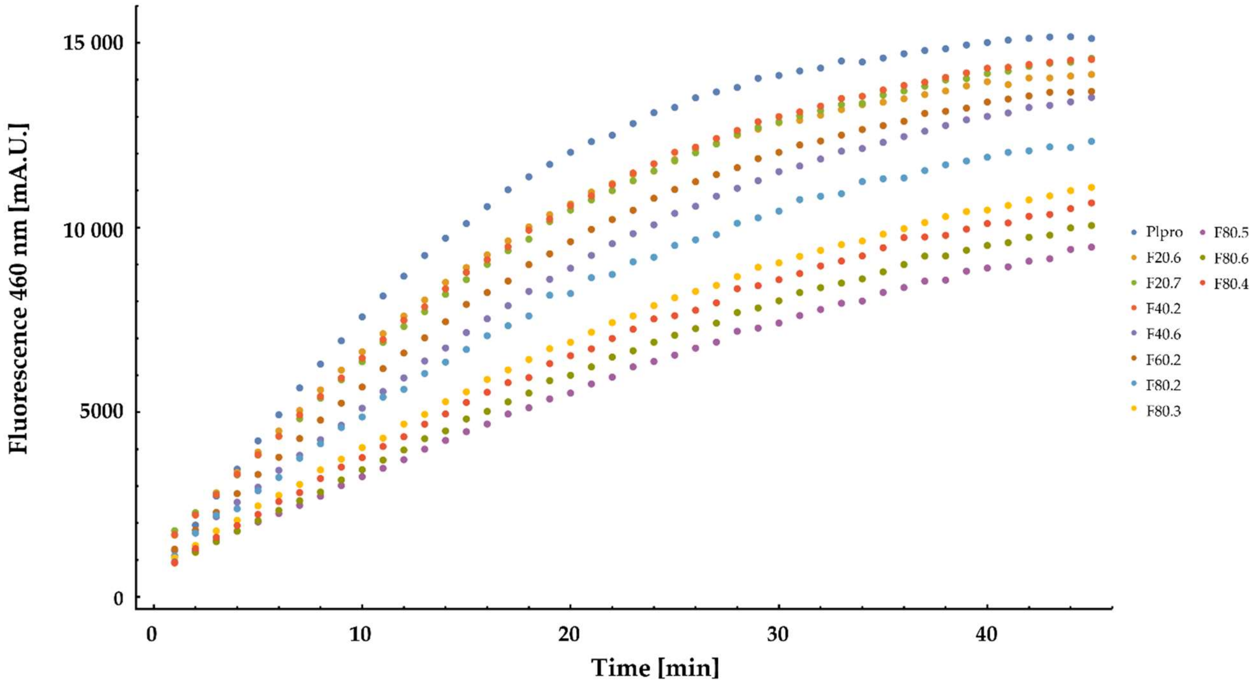

Figure S3. The effects of selected *Pseudanabaena galeata* CCNP1313 flash chromatography fractions on the activity of SARS-CoV-2 PL^{Pro} protease. The fractions are marked as Fx.y, where x stands for MeOH concentration and y is the number of the subsequent fractions eluted at x% MeOH. Data are presented as mean values.

Table S5. The effects of selected *Pseudanabaena galeata* CCNP1313 flash chromatography fractions on the activity of SARS-CoV-2 M^{Pro} protease. inhibition fractions. Data are presented as mean values with standard deviation.

Fraction*	Initial velocity (mAU min^{-1})	SD	Inhibition (%)
M^{Pro}	38	0	0
F40.1	34	2	9
F40.6	33	1	13
F40.7	30	0	22
F60.2	31	0	20
F80.1	29	1	25
F80.2	33	2	14
F80.3	33	2	14
F80.4	29	2	23
F80.5	30	0	21
F80.6	32	0	17

* The fractions are marked as Fx.y, where x stands for MeOH concentration and y is the number of the subsequent fractions eluted at x% MeOH.

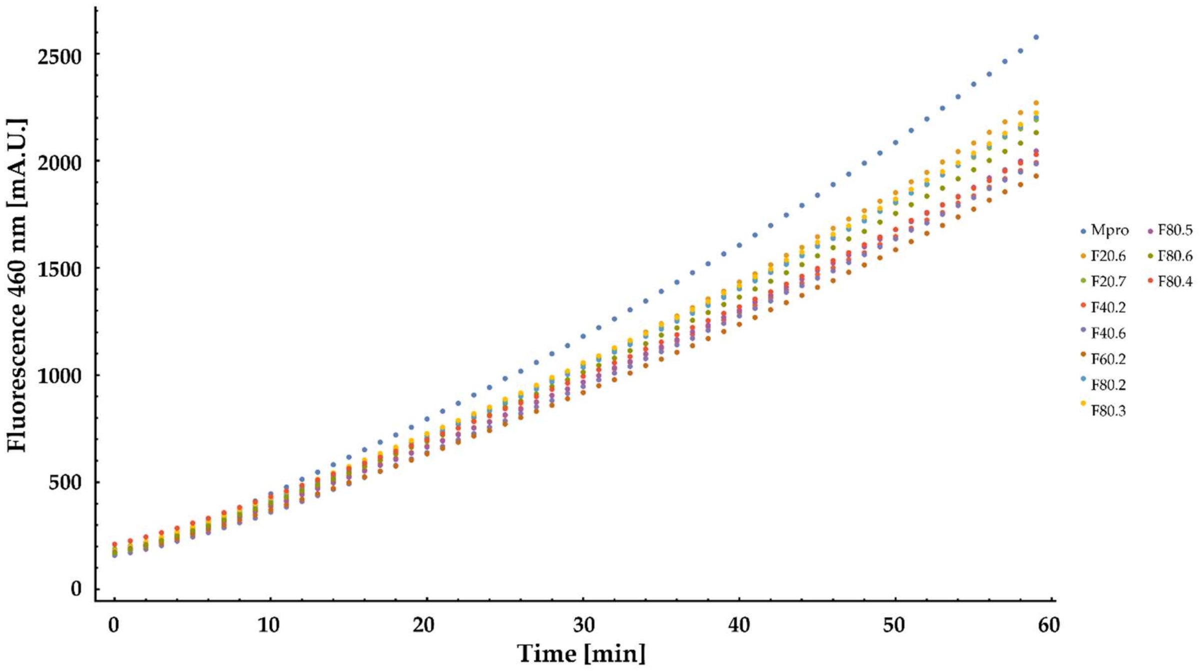

Figure S4. The effects of selected *Pseudanabaena galeata* CCNP1313 flash chromatography fractions on the activity of SARS-CoV-2 M^{Pro} protease. The fractions are marked as Fx.y, where x stands for MeOH concentration and y is the number of the subsequent fractions eluted at x% MeOH. Data are presented as mean values.

Table S6. The effects of isolated peptides and samples containing a mixture of 2–3 peptides from *Pseudanabaena galeata* CCNP1313 on the activity of SARS-CoV-2 PL^{Pro} protease. Data are presented as mean values with standard deviation.

Sample	Initial velocity (mAU min^{-1})	SD	Inhibition (%)
A_1	252	21	19
A_2	205	3	34
A_3	176	34	43
A_4	187	21	40
A_5	202	12	35
A_6	242	18	22
A_7	222	20	28
A_8	227	9	27

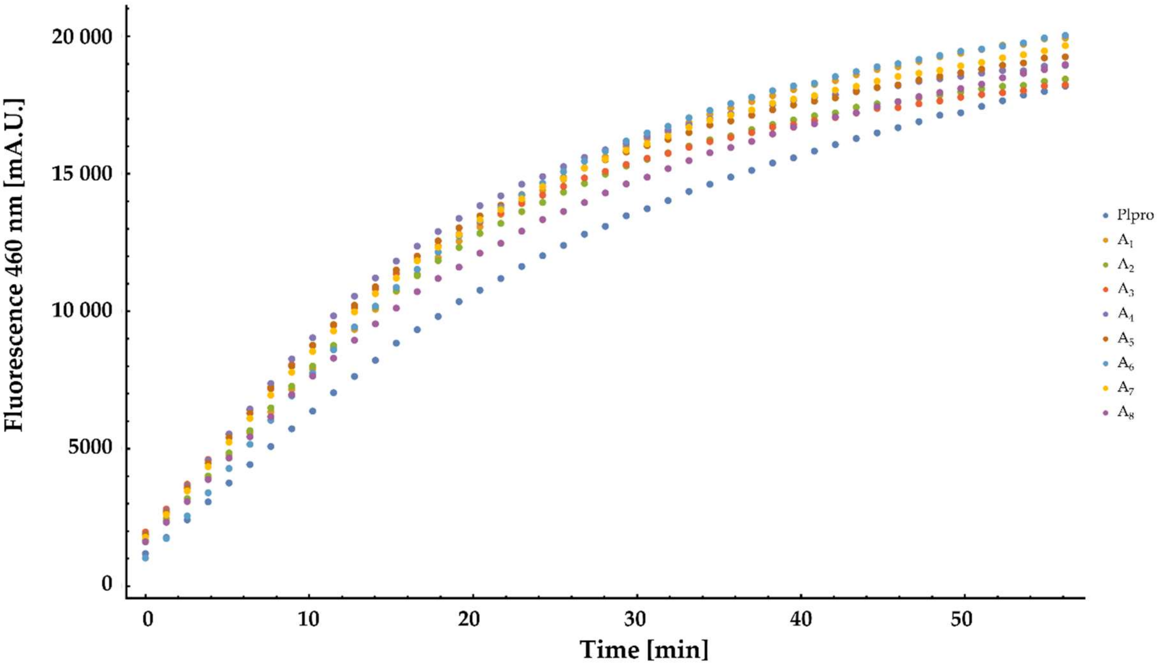

Figure S5. The effects of isolated peptides and samples containing a mixture of 2–3 peptides from *Pseudanabaena galeata* CCNP1313 on the activity of SARS-CoV-2 PL^{Pro} protease.

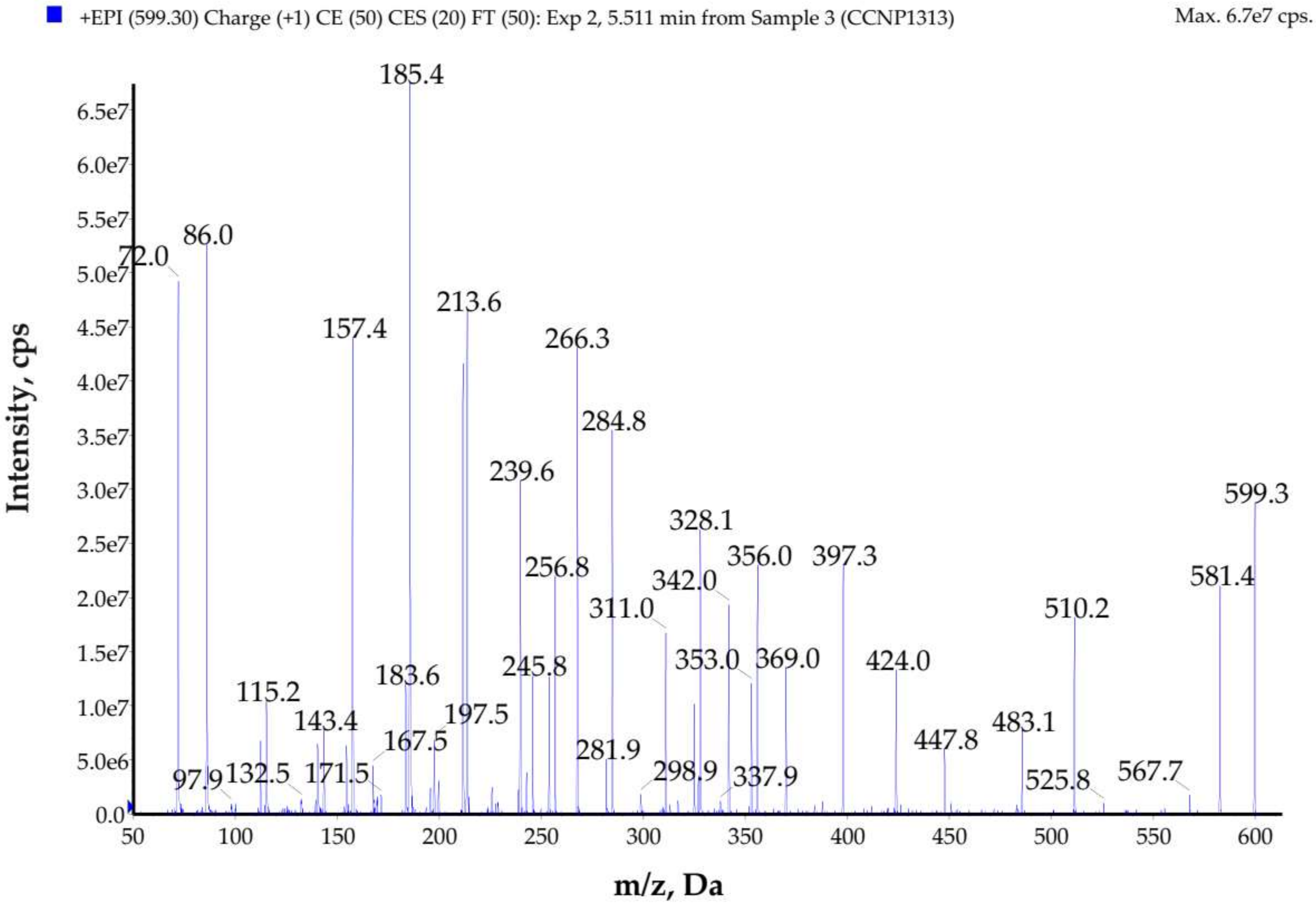

Figure S6. Enhanced product ion mass spectrum of galeapeptin GP598 with the proposed structure Ala-Leu*-Val-Leu*-Leu*-Ala, characterised based on the following ion peaks at *m/z* **599** $[M + H]^+$, 581 $[M + H - H_2O]^+$, 510 $[M + H - Ala^*]^+$, 483 $[M + H - Ala - CO]^+$, 397 $[M + H - (Leu^* + Ala]^+$/$[Ala + Leu^* + Val + Leu^* + H]^+$, 369 $[Ala + Leu^* + Val + Leu^* + H - CO]^+$, 284 $[Ala + Leu^* + Val + H]^+$, 266 $[Ala + Leu^* + Val - H_2O + H]^+$, 256 $[Ala + Leu^* + Val - CO + H]^+$, 213 $[Leu^* + Val + H]^+$, 185 $[Ala + Leu^* + H]^+$/$[Leu^* + Val - CO + H]^+$, 157 $[Leu^* + Ala - CO + H]^+$, 86 Leu* immonium ion, 72 Val immonium ion.

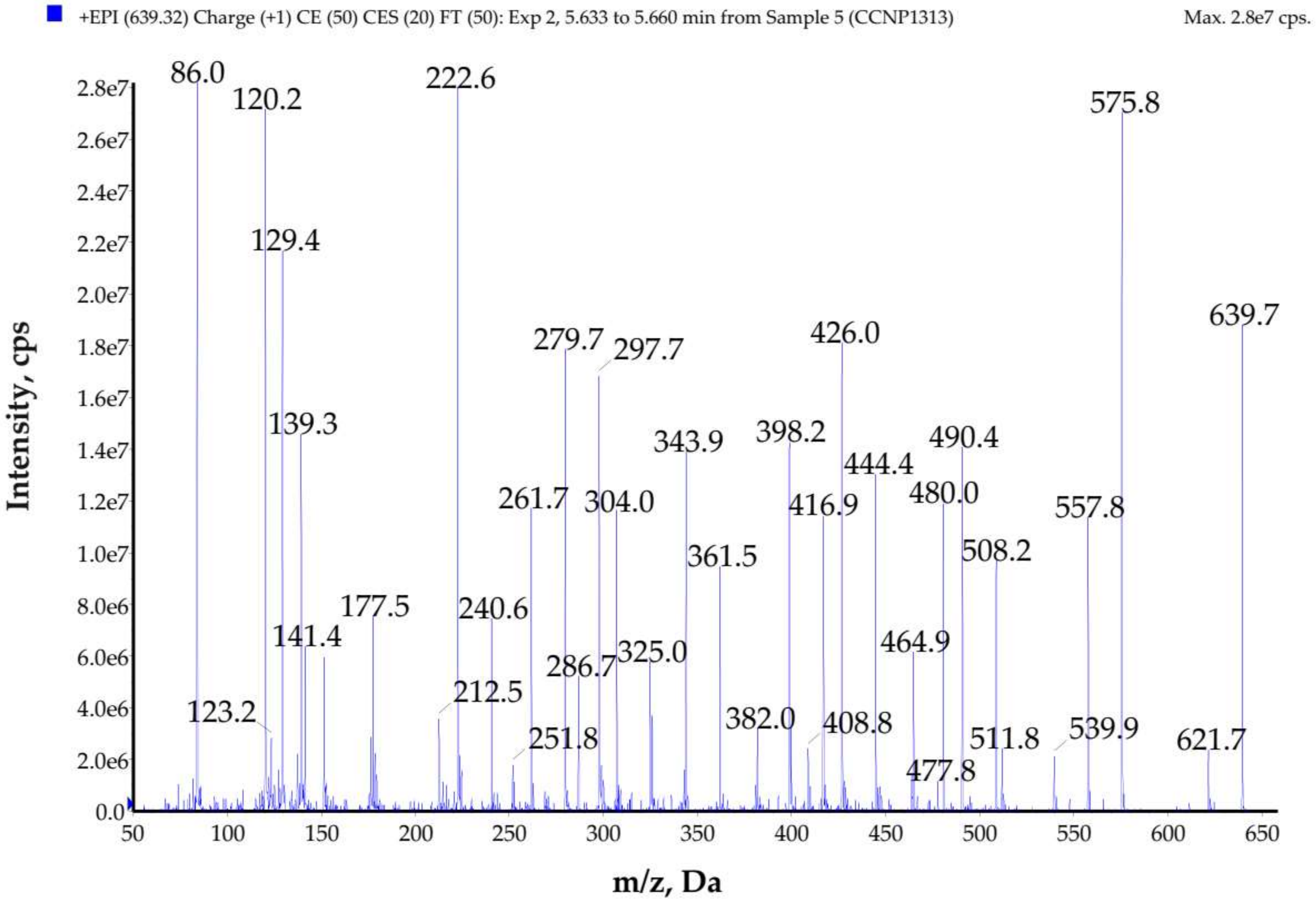

Figure S7. Enhanced product ion mass spectrum of a peptide named PG638 with the proposed structure Arg-MetO-Gly-Phe-Leu*, characterised based on the following ion peaks at *m/z* **639** $[M + H]^+$, 621 $[M + H - H_2O]^+$, 575 $[M + H - CH_3SOH]^+$, 508 $[M + H - Leu^*]^+$, 490 $[M + H - Leu^* - H_2O]^+$, 480 $[M + H - Leu^* - H_2O]^+$, 444 $[M + H - Leu^* - CH_3SOH]^+$, 426 $[M + H - Leu^* - CH_3SOH - H_2O]^+$, 416 $[M + H - Leu^* - CH_3SOH - CO]^+$, 398 $[M + H - Leu^* - CH_3SOH - H_2O - CO]^+$, 361 $[M + H - (Phe + Leu^*)]^+$, 343 $[M + H - (Phe + Leu^*) - H_2O]^+$, 304 $[Arg + Met(O) + H]^+$, 297 $[M + H - CH_3SOH - (Phe + Leu^*)]^+$, 286 $[Arg + Met(O) + H - H_2O]^+$, 279 $[M + H - CH_3SOH - (Phe + Leu^*) - H_2O]^+$, 240 $[Arg + Met(O) + H - CH_3SOH]^+$, 222 $[Arg + Met(O) + H - CH_3SOH - H_2O]^+$, 141 $[Met(O) + Gly + H - CH_3SOH]^+$, 129 Arg immonium ion, 120 Phe immonium ion, 86 Leu* immonium ion.

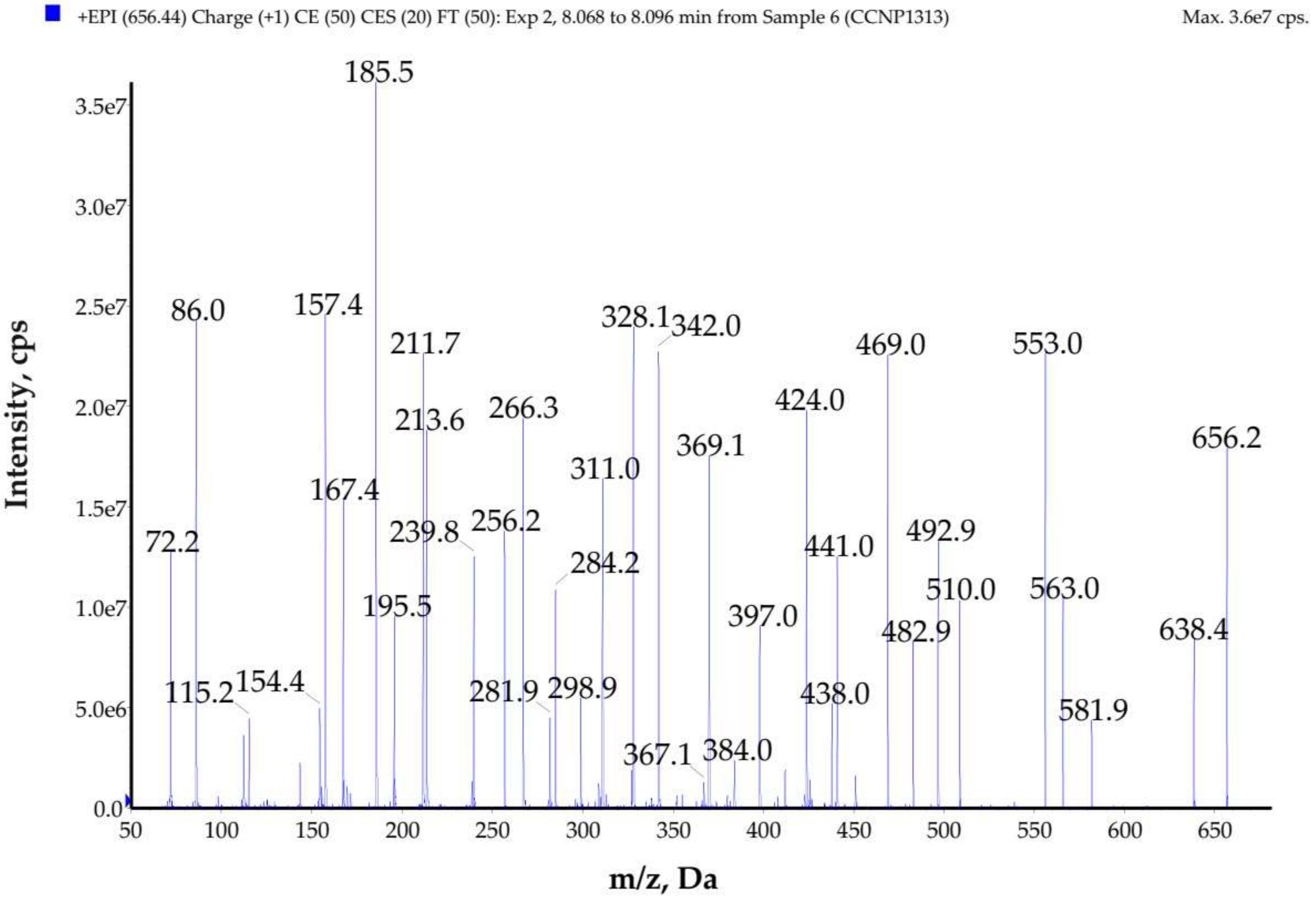

Figure S8. Enhanced product ion mass spectrum of the galeapeptin GP655 with the proposed structure Ala- Leu*-Val-Leu*-Leu*-Ala-Gly, characterised based on the following ion peaks at *m/z* **656** $[M + H]^+$, 638 $[M + H - H_2O]^+$, 581 $[M + H - Gly]^+$, 563 $[M + H - Gly - H_2O]^+$, 553 $[M + H - Gly - CO]^+$, 510 [Ala + Leu* + Val + Leu* + Leu* + H]$^+$, 492 [Ala + Leu* + Val + Leu* + Leu* + H – H_2O]$^+$, 482 [Ala + Leu* + Val + Leu* + Leu* + H – CO]$^+$, 397 [M + H – (Leu* + Ala + Gly)]$^+$/[Ala + Leu* + Val + Leu* + H]$^+$, 369 [Ala + Leu* + Val + Leu* + H – CO]$^+$, 284 [Ala + Leu* + Val + H]$^+$, 266 [Ala + Leu* + Val + H – H_2O]$^+$, 256 [Ala + Leu* + Val + H – CO]$^+$, 213 [Leu* + Val + H]$^+$, 195 [Leu* + Val + H – H_2O]$^+$, 185 [Ala + Leu* + H]$^+$/[Leu* + Val + H – CO]$^+$, 167 [Ala + Leu* + H – H_2O]$^+$, 157 [Leu* + Ala + H – CO]$^+$, 86 Leu* immonium ion, 72 Val immonium ion.

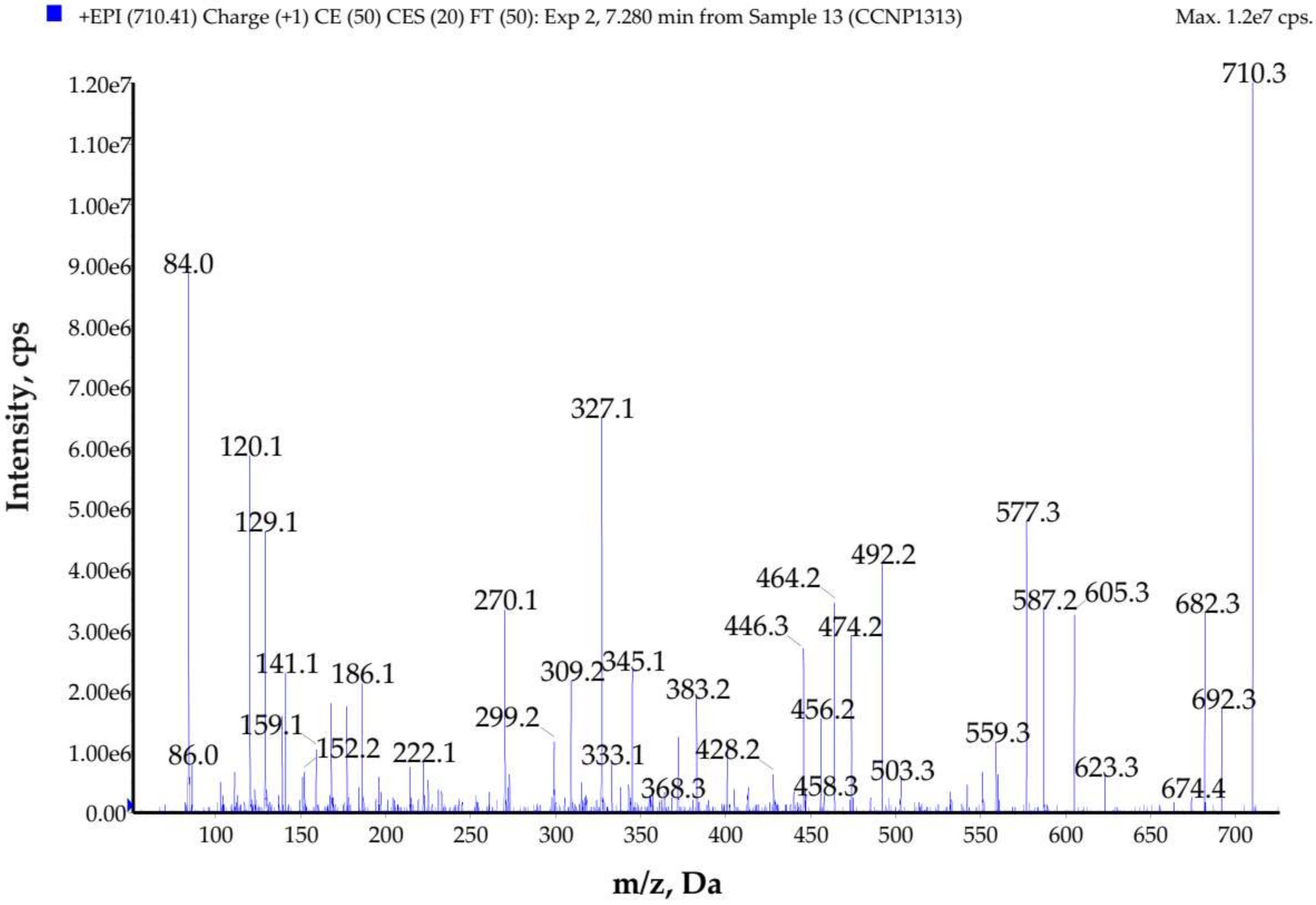

Figure S9. Enhanced product ion mass spectrum of peptide a peptide named PG709 with the proposed structure Arg-Met-Gly-Phe-Leu*-Ser, characterised based on the following ion peaks at *m*/*z* **710** [M + H]+, 692 [M + H – H_2O]+, 682 [M + H – CO]+, 605 [M + H – Ser]+, 587 [M + H – Ser – H_2O]+, 577 [M + H – Ser – CO]+, 559 [M + H – Ser – CO – H_2O]+, 492 [M + H – (Leu* + Ser)]+, 474 [M + H – (Leu* + Ser) – H_2O]+, 464 [M + H – (Leu* + Ser) – CO]+, 446 [M + H – (Leu* + Ser) – H_2O – CO]+, 345 [Arg + Met + Gly + H]+, 327 [Arg + Met + Gly + H – H_2O]+, 270 [Arg + Met + H – H_2O]+, 129 Arg immonium ion, 120 Phe immonium ion, 86 Leu* immonium ion.

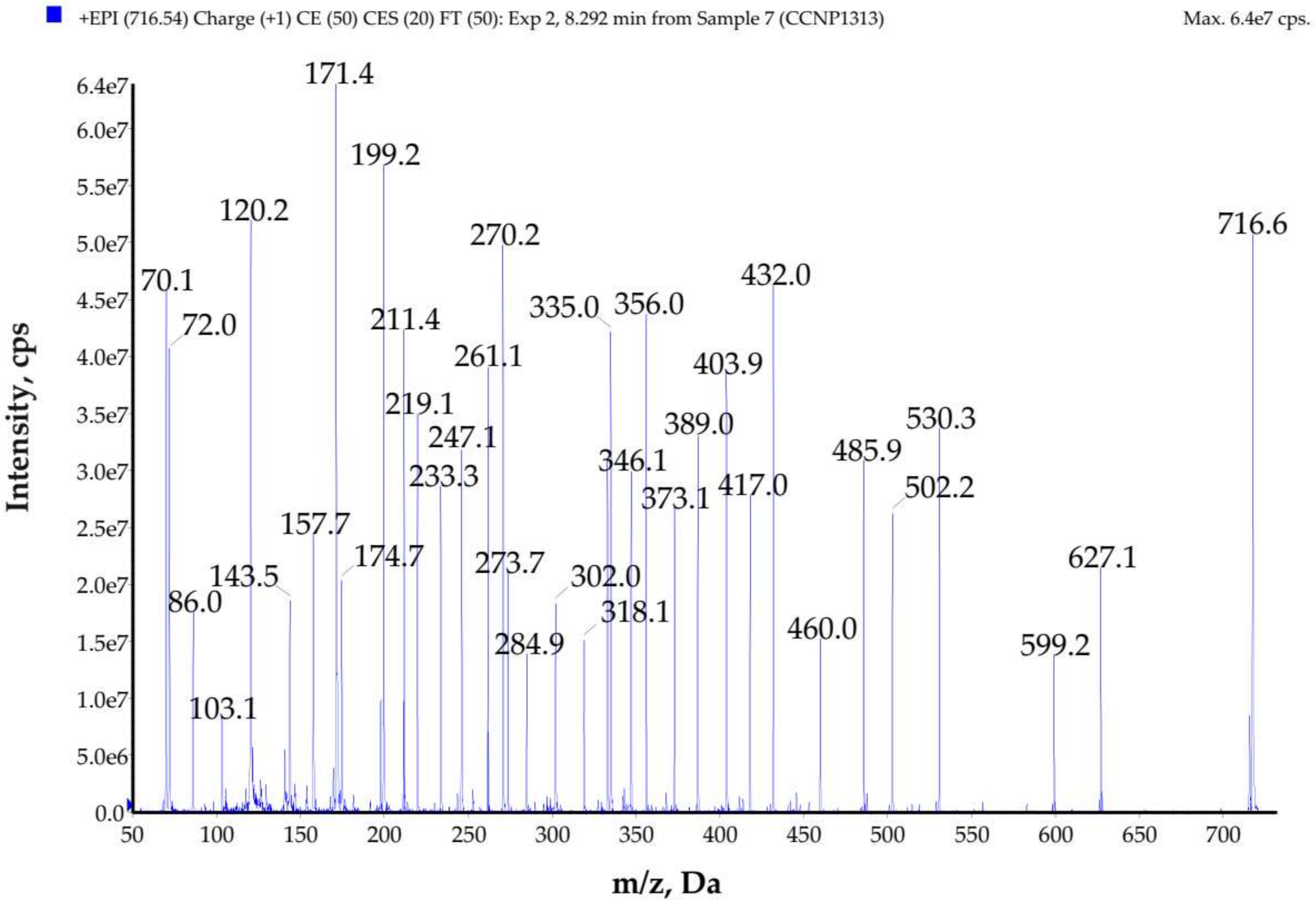

Figure S10. Enhanced product ion mass spectrum of galeapeptin GP715 with the proposed structure Ala-Val-Val-Phe-Leu*-Pro-Ala, characterised based on the following ion peaks at *m/z* **716** [M + H]+, 627 [M + H – Ala]+, 599 [M + H – Ala – CO]+, 530 [M + H – (Pro + Ala)]+, 502 [M + H – (Pro + Ala) – CO]+, 460 [Val + Val + Phe + Leu* + H]+, 432 [Val + Val + Phe + Leu* + H – CO]+, 417 [M + H – (Leu* + Pro + Ala)]+, 389 [M + H – (Leu* + Pro + Ala) – CO]+, 346 [Val + Val + Phe + H]+, 318 [Val + Val + Phe + H – CO]+, 270 [Ala + Val + Val + H]+, 261 [Phe + Leu* + H]+, 247 [Val + Phe + H]+, 233 [Phe + Leu* + H – CO]+, 219 [Val + Phe + H – CO]+, 211 [Leu* + Pro + H]+, 199 [Val + Val + H]+, 171 [Ala + Val + H]+, 143 [Ala + Val + H – CO]+, 120 Phe immonium ion, 86 Leu* immonium ion, 72 Val immonium ion, 70 Pro immonium ion.

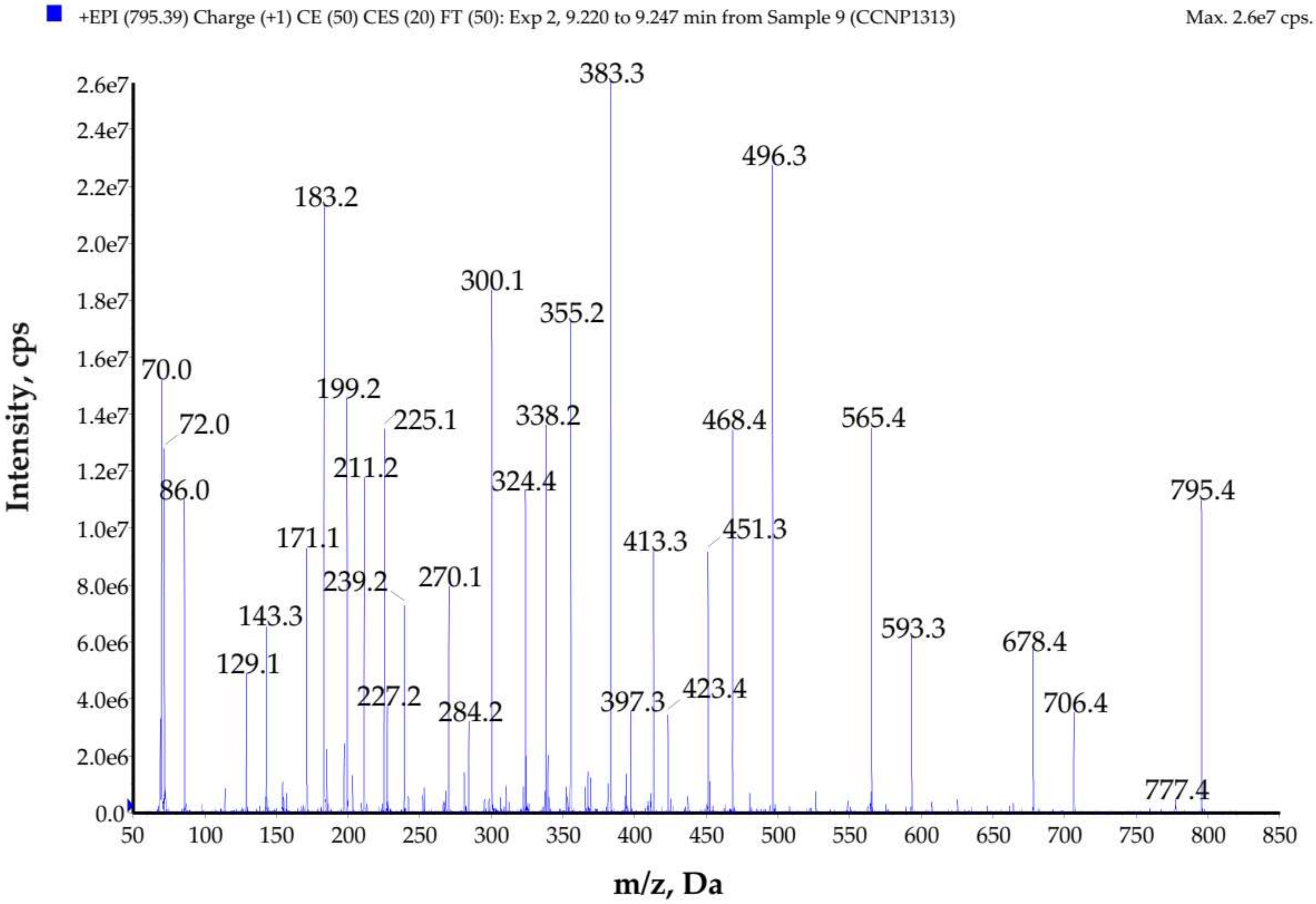

Figure S11. Enhanced product ion mass spectrum of galeapeptin G794 with the proposed structure Ala-Val-Val- Leu*-Leu*-Pro-Leu*-Ala, characterised based on the following ion peaks at *m/z* **795** $[M + H]^+$, 706 $[M + H - Ala]^+$, 678 $[M + H - Ala - CO]^+$, 593 $[M + H - (Leu^* + Ala)]^+$, 565 $[M + H - (Leu^* + Ala) - CO]^+$, 496 $[M + H - (Pro + Leu^* + Ala)]^+$, 468 $[M + H - (Pro + Leu^* + Ala) - CO]^+$, 383 $[Ala + Val + Val + Leu^* + H]^+$, 355 $[Ala + Val + Val + Leu^* + H - CO]^+$, 324 $[Leu^* + Leu^* + Pro + H]^+$, 270 $[Ala + Val + Val + H]^+$, 211 $[Leu^* + Pro + H]^+$, 199 $[Leu^* + Leu^* + H - CO]^+$, 183 $[Leu^* + Pro + H - CO]^+$, 171 $[Ala + Val + H]^+$, 143 $[Ala + Val + H - CO]^+$, 86 Leu* immonium ion, 72 Val immonium ion, 70 Pro immonium ion.

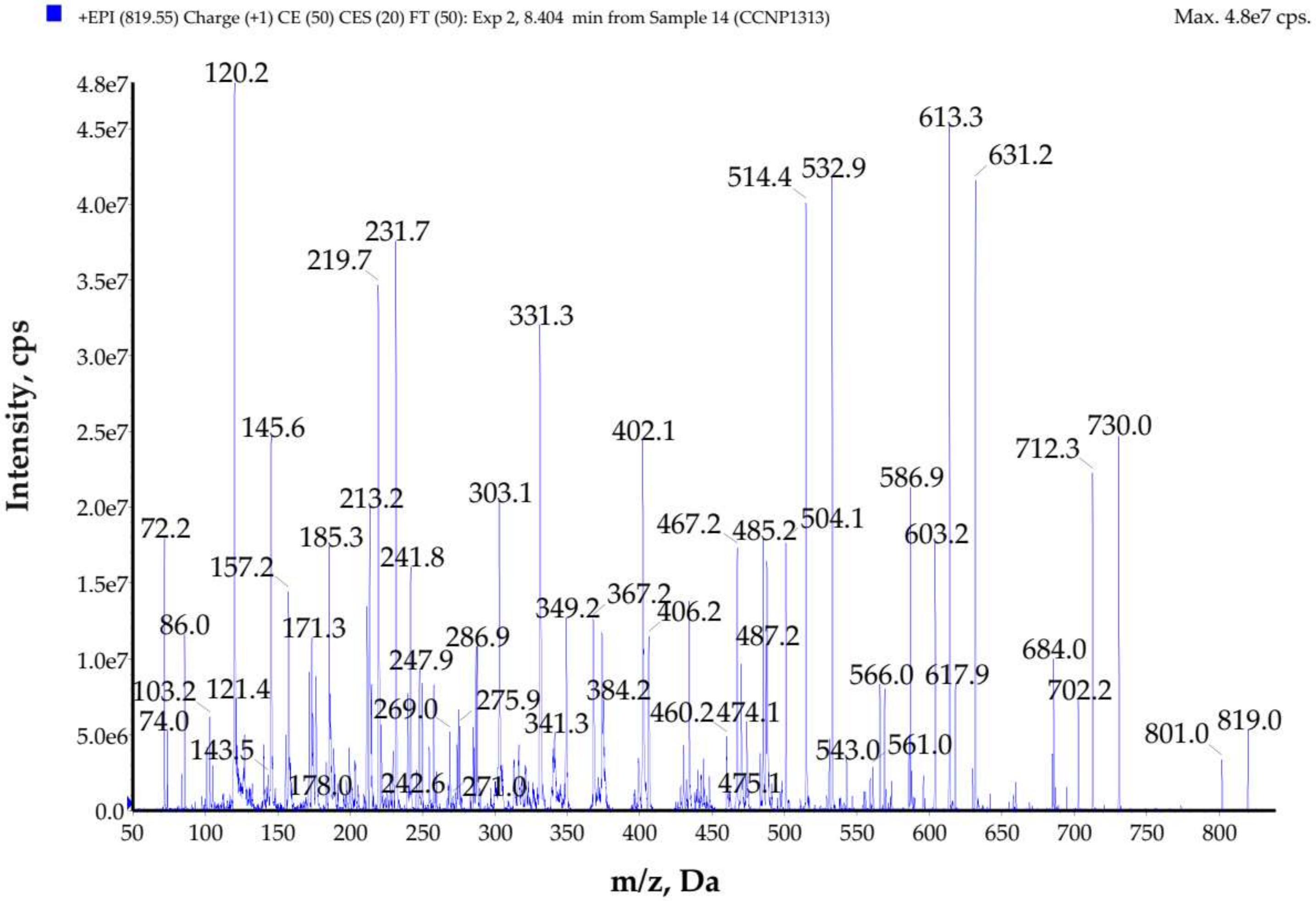

Figure S12. Enhanced product ion mass spectrum of galeapeptin GP818 with the proposed structure Ala-Thr-Leu*-Val-Phe-Val-Val-Ala, characterised based on the following ion peaks at *m/z* **819** $[M + H]^+$, 801 $[M + H - H_2O]^+$, 730 $[M + H - Ala]^+$, 712 $[M + H - Ala - H_2O]^+$, 702 $[M + H - Ala - CO]^+$, 684 $[M + H - Ala - H_2O - CO]^+$, 631 $[M + H - (Val + Ala)]^+$, 613 $[M + H - (Val + Ala) - H_2O]^+$, 603 $[M + H - (Val + Ala) - CO]^+$, 532 $[M + H - (Val + Val + Ala)]^+$, 514 $[M + H - (Val + Val + Ala) - H_2O]^+$, 504 $[M + H - (Val + Ala) - CO]^+$, 367 $[M + H - (Phe - Val + Val + Ala) - H_2O]^+$, 213 $[Leu^* + Val + H]^+$, 185 $[Ala + Leu^* + H]^+$, 157 $[Ala + Leu^* + H - CO]^+$, 171 $[Val + Val + H - CO]^+$, 120 Phe immonium ion, 86 Leu* immonium ion, 74 Thr immonium ion, 72 Val immonium ion.

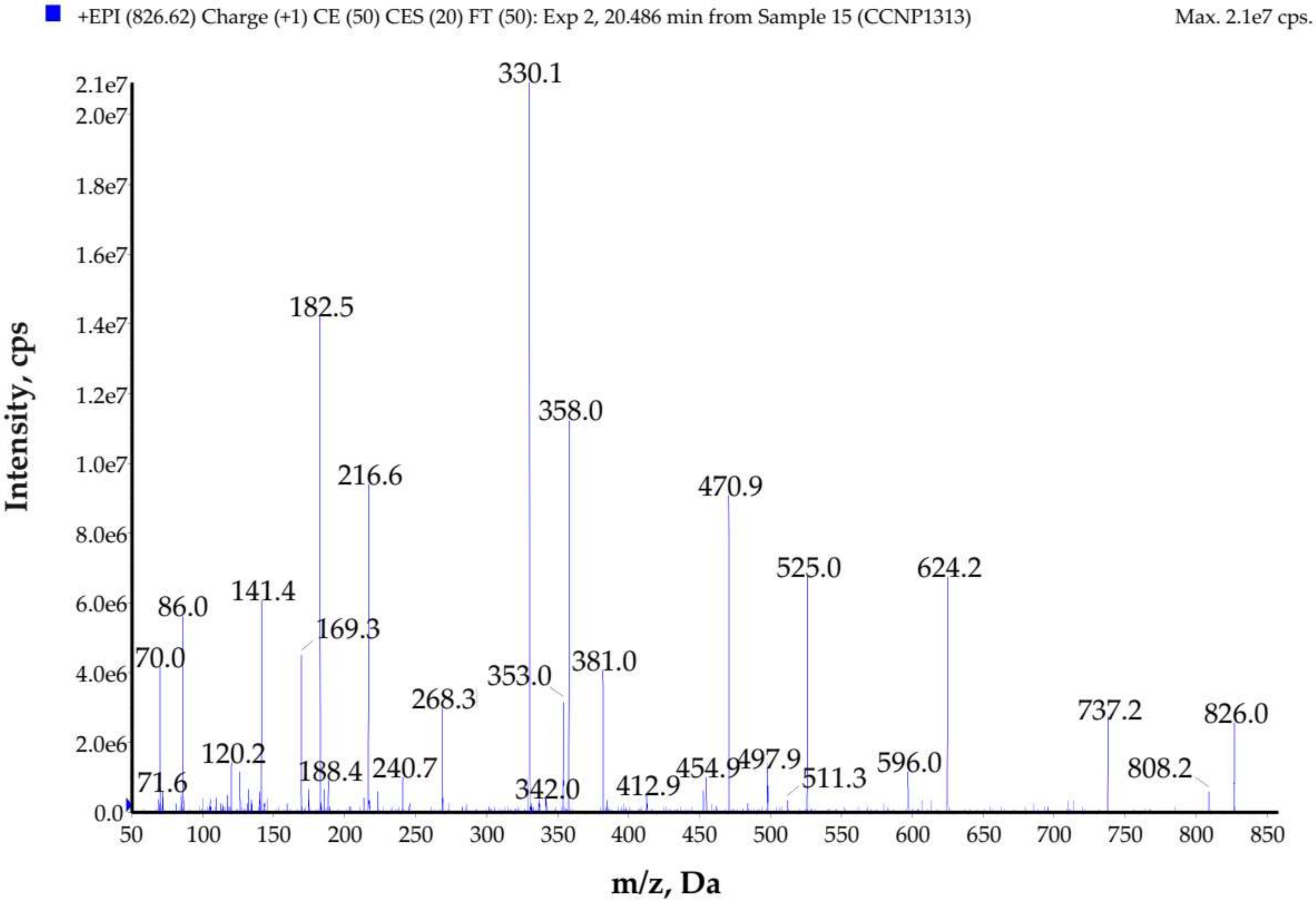

Figure S13. Enhanced product ion mass spectrum of galeapeptin GP825 with the proposed partially elucidated structure Ala-Pro-Val-Leu*-?-Val-Leu*-Ala, characterised based on the following ion peaks at *m*/*z* **826** $[M + H]^+$, 808 $[M + H - H_2O]^+$, 737 $[M + H - Ala]^+$, 624 $[M + H - (Leu^* + Ala)]^+$, 596 $[M + H - (Leu^* + Ala) - CO]^+$, 525 $[M + H - (Val + Leu^* + Ala)]^+$, 497 $[M + H - (Val + Leu^* + Ala) - CO]^+$, 381 $[Ala + Pro + Val + Leu^* + H]^+$, 353 $[Ala + Pro + Val + Leu^* + H - CO]^+$, 268 $[Ala + Pro + Val + H]^+$, 169 $[Ala + Pro + H]^+$, 141 $[Ala + Pro + H - CO]^+$, 120 Phe immonium ion, 86 Leu* immonium ion.

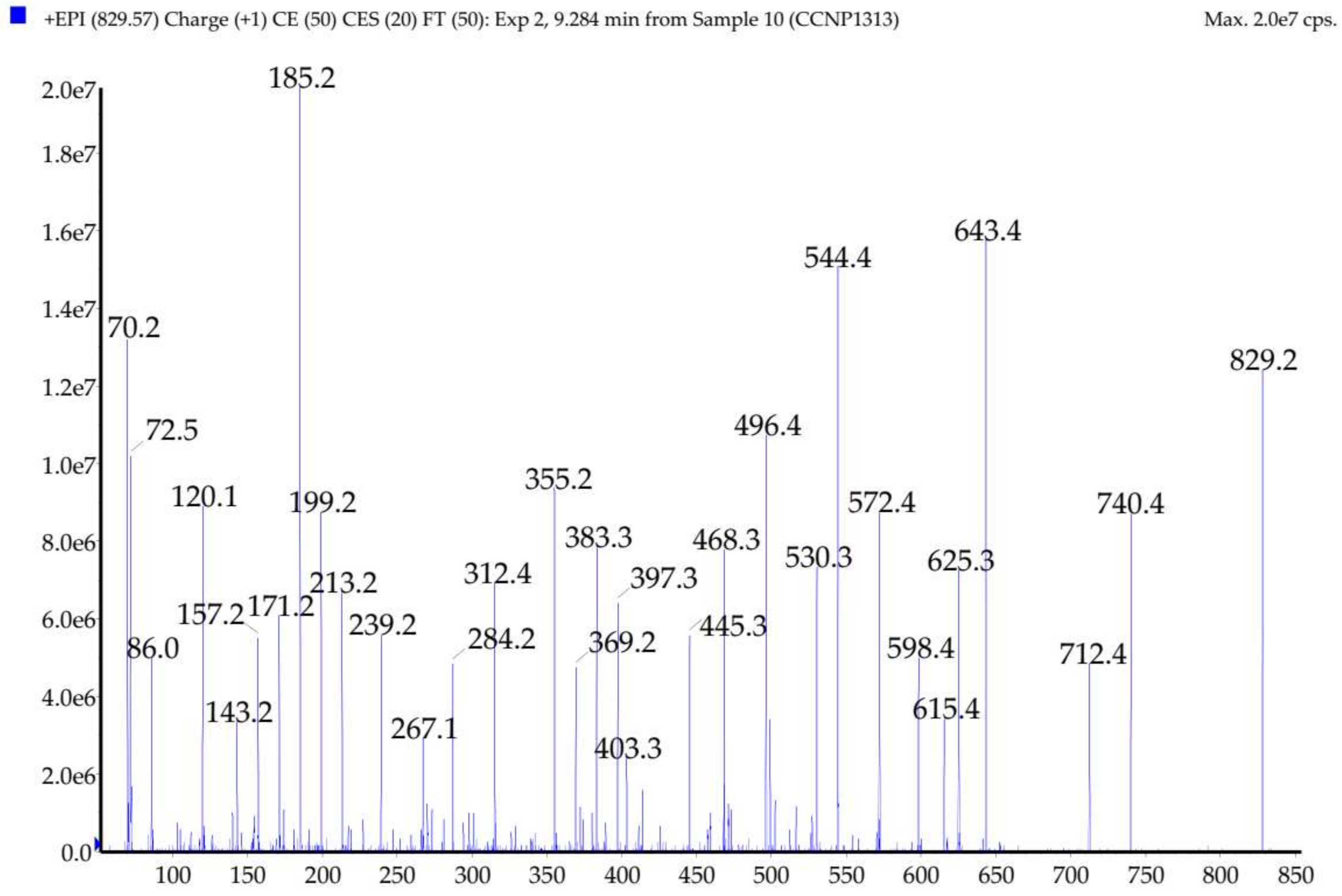

Figure S14. Enhanced product ion mass spectrum of galeapeptin GP828 with the proposed structure Ala-Leu*-Val-Val-Leu*-Phe-Pro-Ala, characterised based on the following ion peaks at *m/z* **829** [M + H]+, 740 [M + H – Ala]+, 712 [M + H – Ala – CO]+, 643 [M + H – (Pro + Ala)]+, 625 [M + H – (Pro + Ala) – H_2O]+, 615 [M + H – (Pro + Ala) – CO]+, 572 [M + H – Ala – (Pro + Ala)]+, 544 [M + H – Ala – (Pro + Ala) – CO]+, 496 [M + H – (Pro + Ala)]+, 468 [M + H – (Pro + Ala) – CO]+, 383 [Ala + Leu* + Val + Val + H]+, 355 [Ala + Leu* + Val + Val + H – CO]+, 312 [Leu* + Val + Val + H]+, 284 [Ala + Leu* + Val + H]+, 213 [Leu* + Val + H]+, 185 [Leu* + Val + H – CO]+/ [Ala + Leu* + H]+, 199 [Val + Val + H]+, 157 [Ala + Leu* + H – CO]+, 171 [Val + Val + H – CO]+, 120 Phe immonium ion, 86 Leu* immonium ion, 72 Val immonium ion, 70 Pro immonium ion.

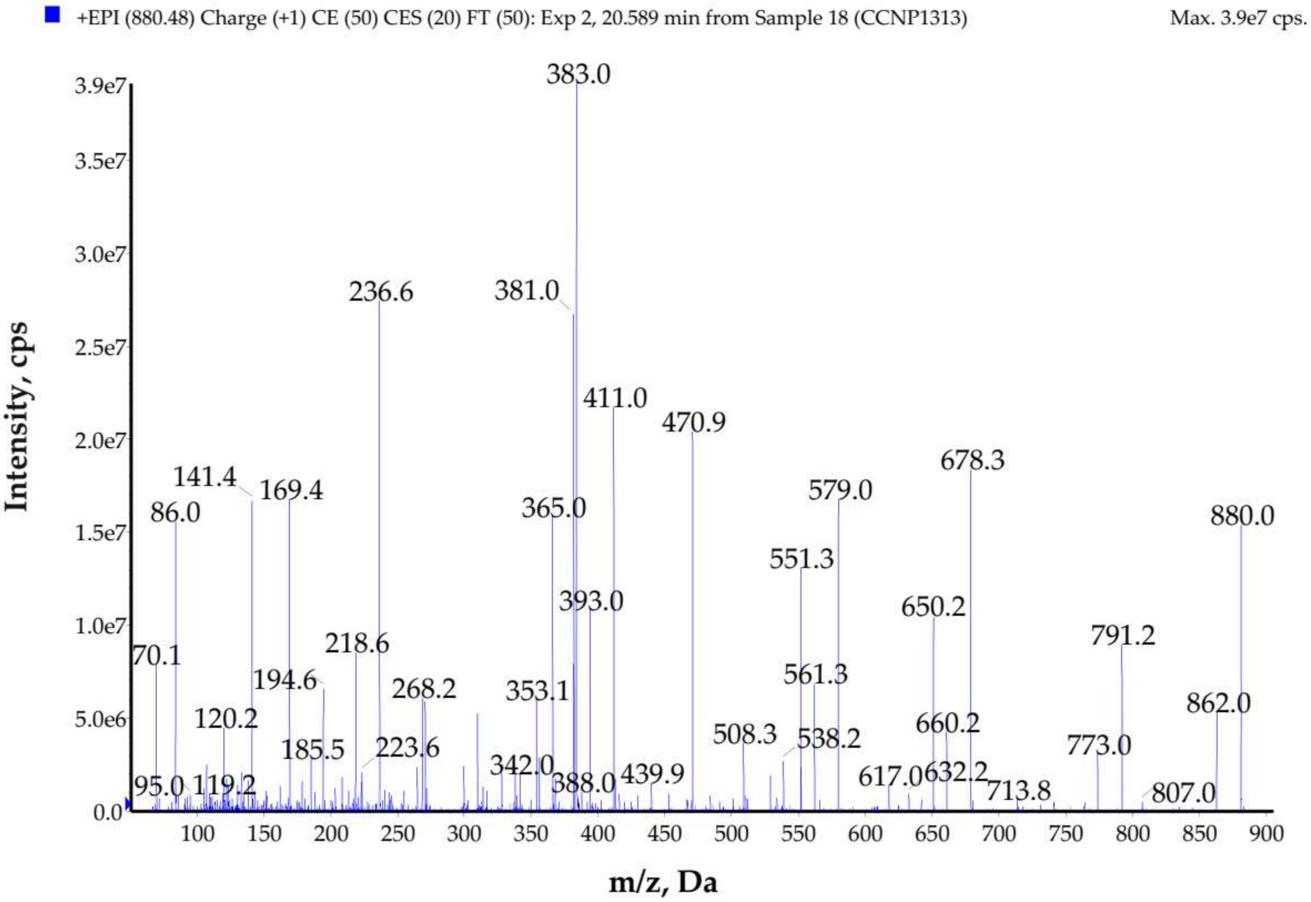

Figure S15. Enhanced product ion mass spectrum of galeapeptin GP879 with the proposed partially elucidated structure Ala-Pro-Val-?-Pro-Ala-Val-Leu*-Ala, characterised based on the following ion peaks at *m*/*z* **880** $[M + H]^+$, 862 $[M + H - H_2O]^+$, 791 $[M + H - Ala]^+$, 773 $[M + H - Ala - H_2O]^+$, 678 [M + H – (Leu* + Ala)]$^+$, 660 [M + H – (Leu* + Ala) – H_2O]$^+$, 650 [M + H – (Leu* + Ala) – CO]$^+$, 632 [M + H – (Leu* + Ala) – H_2O – CO]$^+$, 579 [M + H – (Val + Leu* + Ala)]$^+$, 561 [M + H – (Val + Leu* + Ala) – H_2O]$^+$, 551[M + H – (Val + Leu* + Ala) – H_2O – CO]$^+$, 508 [M + H – (Ala + Val + Leu* + Ala)]$^+$, 411 [M + H – (Pro + Ala + Val + Leu* + Ala)]$^+$, 393 [M + H – (Pro + Ala + Val + Leu* + Ala) – H_2O]$^+$, 383 [M + H – (Pro + Ala + Val + Leu* + Ala) – CO]$^+$, 381 [Ala + Pro + Val + Leu* + H]$^+$, 365 [M + H – (Pro + Ala + Val + Leu* + Ala) – H_2O – CO]$^+$, 353 [Ala + Pro + Val + Leu* + H – CO]$^+$, 268 [Ala + Pro + Val + H]$^+$, 169 [Ala + Pro + H]$^+$, 141 [Ala + Pro + H – CO]$^+$, 120 Phe immonium ion, 86 Leu* immonium ion.

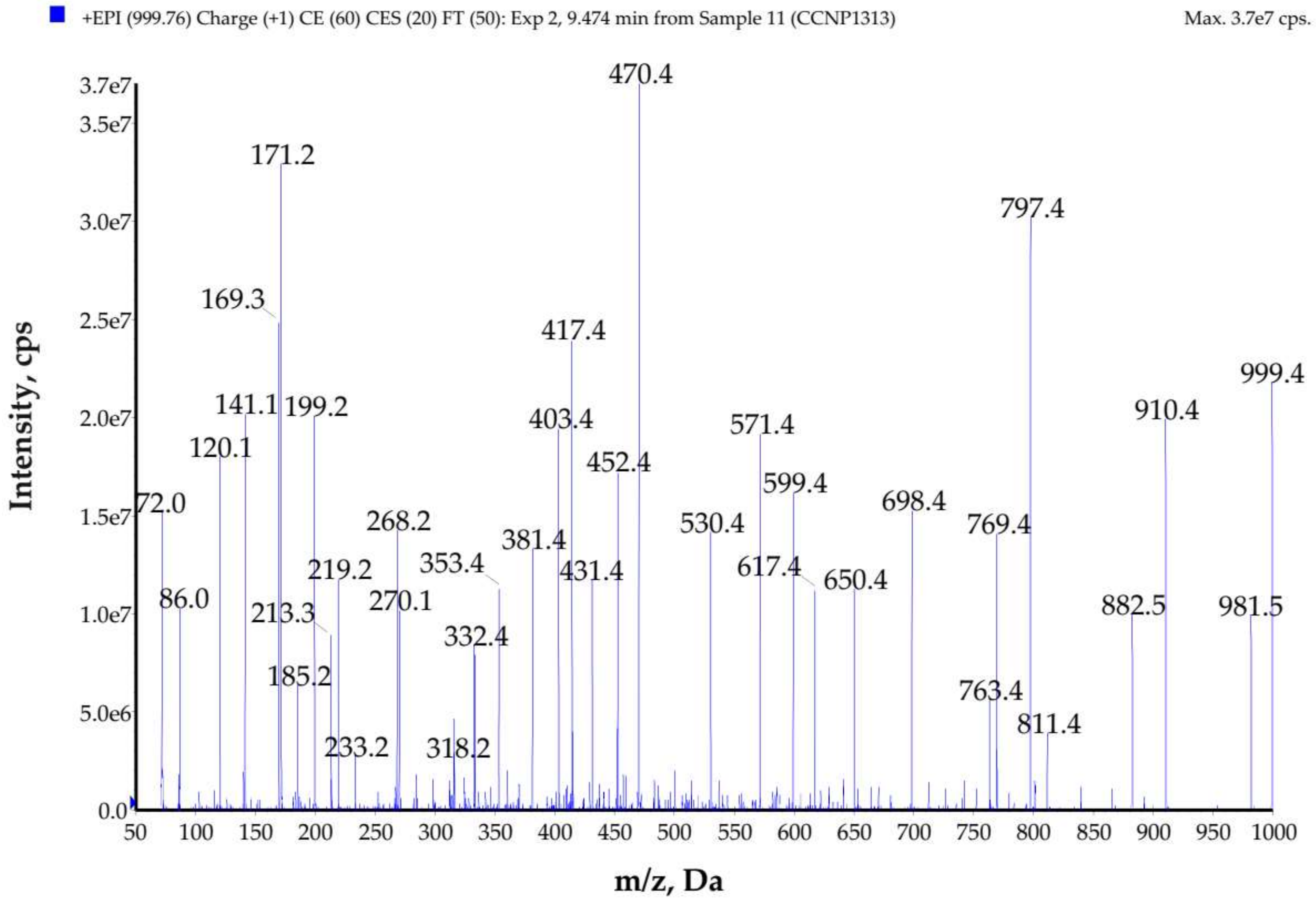

Figure S16. Enhanced product ion mass spectrum of galeapeptin GP998 with the proposed structure Ala-Pro-Val-Leu*-Ala-Phe-Val-Val-Leu*-Ala, characterised based on the following ion peaks at *m*/*z* **999** [M + H]+, 981 [M + H – H_2O]+, 910 [M + H – Ala*]+, 882 [M + H – Ala – CO]+, 797 [M + H – (Leu* + Ala)]+, 769 [M + H – (Leu* + Ala) – CO]+, 698 [M + H – (Val + Leu* + Ala)]+, 599 [M + H – (Val + Val + Leu* + Ala)]+, 571 [M + H – (Val + Val + Leu* + Ala) – CO]+, 530 [Val + Leu* + Ala + Phe + Val + H]+, 452 [Ala + Pro + Val + Leu* + Ala + H]+, 431 [Val + Leu* + Ala + Phe + H]+, 417 [Ala + Phe + Val + Val +H]+, 403 [Val + Leu* + Ala + Phe + H – CO]+, 381 [Ala + Pro + Val + Leu* + H]+, 353 [Ala + Pro + Val + Leu* + H - CO]+, 332 [Leu* + Ala + Phe + H]+, 268 [Ala + Pro + Val + H]+, 219 [Ala + Phe + H]+, 213 [Val + Leu* + H]+, 199 [Val + Val + H]+, 185 [Val + Leu* – CO + H]+, 171 [Val + Val + H – CO]+, 169 [Ala + Pro + H]+, 141 [Ala + Pro + H – CO]+, 120 Phe immonium ion, 86 Leu* immonium ion, 72 Val immonium ion.

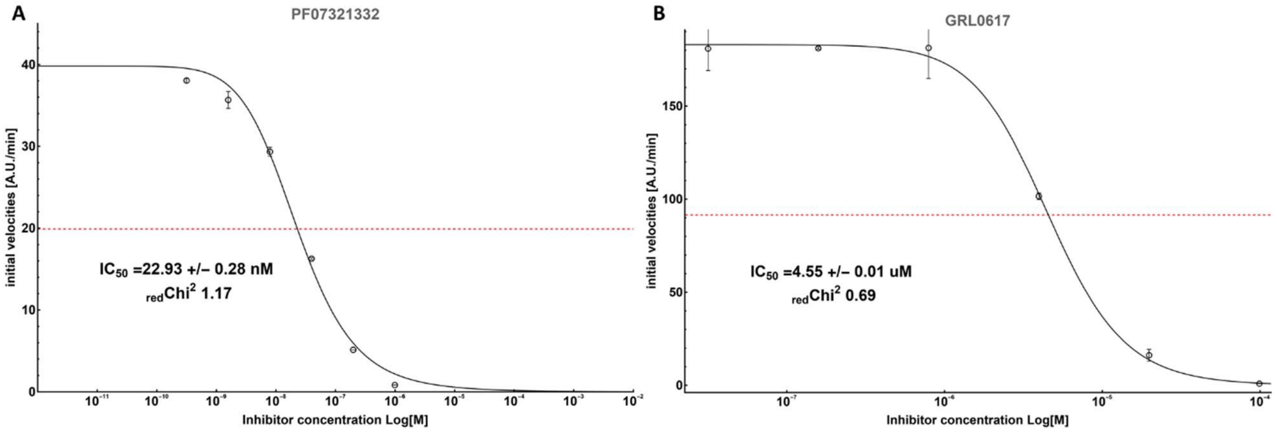

Figure S17. Positive controls and IC_{50} values determination for enzymatic assays (**A**) M^{pro} (IC_{50} for PF-07321332 a well-known M^{pro} inhibitor, commercial name nirmatrelvir with reported literature IC_{50} value of 19.2 nM) and (**B**) PL^{pro} (IC_{50} determination for GRL-0617, a well-known PL^{pro} inhibitor with reported literature IC_{50} value of 2.1 μM).
